# Physiological and Multi-Omics Insights into *Trichoderma harzianum* Alleviating Aged Microplastic Stress in *Nicotiana benthamiana*

**DOI:** 10.3390/ijms262311667

**Published:** 2025-12-02

**Authors:** Feiyan Wang, Xiaoyan Sun, Ke Wang, Bibo Long, Fayong Li, Dong Xie

**Affiliations:** 1 Institute of Biological and Medical Engineering, Guangdong Academy of Sciences, Guangzhou 510316, China; wfei08@163.com (F.W.);; 2 Guangdong Biomaterials Engineering Technology Research Center, Guangzhou 510316, China

**Keywords:** *Nicotiana benthamiana*, *Trichoderma harzianum*, aged microplastics, transcriptome, soil microbiome

## Abstract

Microplastics derived from biodegradable PBAT film, widely used in agriculture, pose ecological and biological hazards. This study explores how *Trichoderma harzianum* T4 mitigates this microplastic-induced stress in *Nicotiana benthamiana*. Using five experimental setup-control (CK), low/high-dose aged microplastics (MP80/MP320), and their co-treatments with *T. harzianum* T4 (MP80+T4/MP320+T4), multi-omics analyses reveal the microplastic stress-alleviating mechanisms of *T. harzianum* T4. Aged microplastics significantly inhibit plant growth, promote reactive oxygen species (ROS) and malondialdehyde (MDA) accumulation, and disrupt metabolic homeostasis. Conversely, *T. harzianum* T4 activates the plant antioxidant defense system, reducing ROS/MDA levels and upregulating superoxide dismutase (SOD)/peroxidase (POD) activities, and promotes biomass. Transcriptomic analysis shows *T. harzianum* T4 reverses gene expression patterns disrupted by microplastics, particularly in DNA replication and pentose–glucuronic acid pathways. Metagenomic sequencing indicates *T. harzianum* T4 restores soil microbial diversity, increases the abundance of *Bacteroidota* and *Myxococcota*, downregulates antibiotic resistance genes (e.g., *tetA5*, *MDR*), and upregulates carbohydrate-active enzymes (CAZys), thereby enhancing carbon metabolism. In conclusion, *T. harzianum* T4 alleviates microplastic stress through a tripartite mechanism: activating plant stress-response gene networks, reshaping soil microbial communities, and modulating functional gene expression, offering a promising bioremediation strategy.

## 1. Introduction

Plastics are widely used in industry, agriculture, and daily life due to their superior properties (e.g., lightweight, durability, cost-effectiveness, malleability) [1,2,3], but their extensive use has led to global environmental pollution. Microplastics, emerging pollutants defined as particles < 5 mm, originate from plastic waste via natural physical and chemical degradation [2]. Microplastics can be categorized into persistent and biodegradable types. And both accumulate in soils to threaten soil ecosystems and food security, with adverse effects on marine environments [4,5], agricultural planting environments, and human health [6,7,8]. For crops, microplastics impair seed germination [9], root/aboveground growth [10,11], and nutrient uptake [12]. Specifically, PBAT-based microplastics (PBAT-MPs) reduce root length, surface area, and biomass in soybean and maize [13], while PVC microplastics (PVC-MPs) inhibit rice growth and biomass accumulation [14]. Higher microplastic doses can alter crop water use efficiency, internal CO_2_ levels [14], antioxidant systems [15], osmoregulatory substances, hormone balance, and nutrient transport [16,17]. Notably, biodegradable plastic residues (macro- and micro-sized) exert stronger negative effects on wheat growth and earthworm survival than polyethylene residues [18], highlighting the need for further degradation of biodegradable microplastics to ensure ecological friendliness and food safety.

In the field of plants, microplastics modify soil enzyme activity, microbial communities, and functional genes, thereby disrupting biogeochemical cycling of carbon, nitrogen, and phosphorus [19]. Plant responses to microplastic stress are concentration dependent. Low doses may enhance aboveground biomass, while high doses promote belowground biomass allocation as an adaptive strategy [20]. Microplastics also induce oxidative stress [21], reduce photosynthesis (12% in terrestrial plants, 7% in marine algae [22]), and alter gene expression in pathways like phenylpropanoid biosynthesis and sucrose metabolism of cabbage [23] and in the roots of maize seedlings [24]. Nanoplastics downregulate antioxidant-related genes, leading to hydrogen peroxide accumulation and growth inhibition [25]. Beyond single-gene changes, microplastic stress reshapes complex plant gene regulatory networks by activating transcription factors (e.g., APX1, WRKYs) that modulate antioxidant enzyme (SOD, POD, CAT) expression [26] and disrupting nitrogen/phosphorus metabolism [27,28]. For biodegradable microplastics, they reduce soil microbial diversity, inhibit carbon–nitrogen–phosphorus cycle-related enzymes [29], and increase soil antibiotic resistance genes (ARGs), with multidrug resistance genes becoming dominant [30]. Therefore, the genetic response of plants to microplastic stress is not merely isolated changes in single genes but rather involves the complex reshaping of gene regulatory networks [31].

Microorganisms play a vital role in ecological cycling, human health, and industrial and agricultural development, serving as significant factors in nature [32]. Microorganisms also help mitigate microplastic pollution and crop stress. When addressing microplastic stress, *Pseudomonas aeruginosa* capture PS-NPs via extracellular polymers to reduce plant uptake [33], while rhizosphere microbes utilize PHBV as a carbon source to enhance microbial biomass in microplastic-contaminated soils [29]. *Lactobacillus paracasei* DT66 and *Lactobacillus plantarum* DT88 adsorb diverse microplastics (PS, PE, PC, PP, PET) and increase the excretion of MPs in animals [34]. Bacteria can secrete extracellular enzymes that act on the surface of microplastics, facilitating degradation or modification. *Exiguobacterium marinum* a-1 can effectively degrade polypropylene films [35]. *Trichoderma harzianum*, a ubiquitous filamentous fungus, promotes plant growth, enhances stress resistance, and exhibits biodegradative potential. It degrades polyethylene at a 40% rate (higher than sterilization controls) via laccase and manganese peroxidase [36,37]. And combined with *Aspergillus niger*, *T. harzianum* fungi induces significant physical changes in PET, such as color changes, surface curvature, and scratches [38]. However, the mechanisms by which *T. harzianum*-derived enzymes degrade microplastics and alleviate plant stress remain poorly understood, requiring further validation (e.g., enzyme activity assays, degradation product analysis).

This study aims to validate the degradation capacity of *T. harzianum* T4, a PBAT-degrading microorganism isolated from PBAT plastic films, and decipher its regulatory mechanisms on plant growth under microplastic stress. Using *N. benthamiana* as the model plant, multiple interaction experiments were conducted to quantify the stress impacts of PBAT microplastics on plant growth and the alleviative effects of *T. harzianum* T4 against microplastic stress through integrative analysis of agronomic traits, transcriptomics, and soil metagenomics of *N. benthamiana*. These findings will provide critical theoretical insights for addressing microplastic pollution and harnessing the functional potentials of *T. harzianum* in ecological remediation.

## 2. Results

### 2.1. Mycelial Characteristics and Molecular Identification of T. harzianum

**Figure 1:**
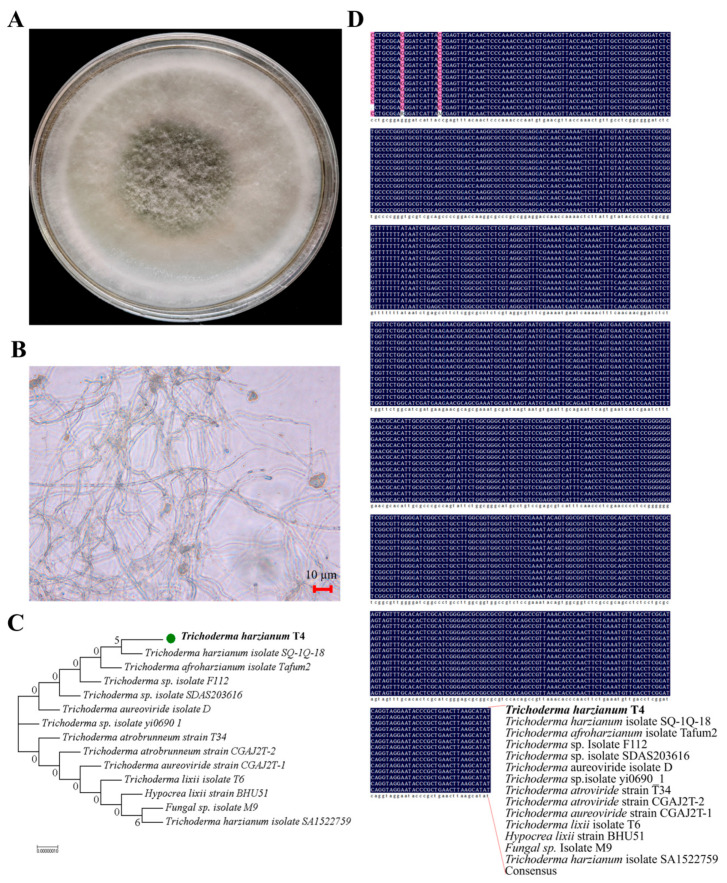
*T. harzianum* T4 colony morphology and phylogenetic tree. (**A**) Colony morphology of *T. harzianum* T4 on PDA medium. (**B**) Mycelial microstructure of *T. harzianum* T4 under optical microscope. (**C**) Phylogenetic tree of *T. harzianum* T4 based on ITS sequence. (**D**) ITS sequence alignment of *T. harzianum* T4 with reference strains.

In this study, the mycelial characteristics of the functional fungal strain were systematically analyzed. The strain can grow well on potato dextrose agar (PDA) medium at 28 °C (Figure 1A). Microscopic observation showed that the mycelia were septate structures, with a hyaline appearance and branched growth (Figure 1B). For molecular identification, PCR amplification of the internal transcribed spacer (ITS) region generated specific DNA fragments of expected sizes. Sequencing and subsequent BLAST (2.17.0) analysis confirmed that the isolated strain was *Trichoderma harzianum*, displaying ≥99% sequence similarity to GenBank reference sequences, and was designated as *Trichoderma harzianum* isolate T4 (Figure 1). Phylogenetic analysis based on combined ITS sequences distinguished *Trichoderma harzianum* T4 from closely related *Trichoderma* species (e.g., *Trichoderma harzianum* isolate SQ-1Q-18), providing robust molecular evidence for its taxonomic identification (Figure 1B–D). These results provide detailed insights into the mycelial traits and taxonomic status of this strain.

### 2.2. Degradation of Film Properties by T. harzianum T4

**Figure 2:**
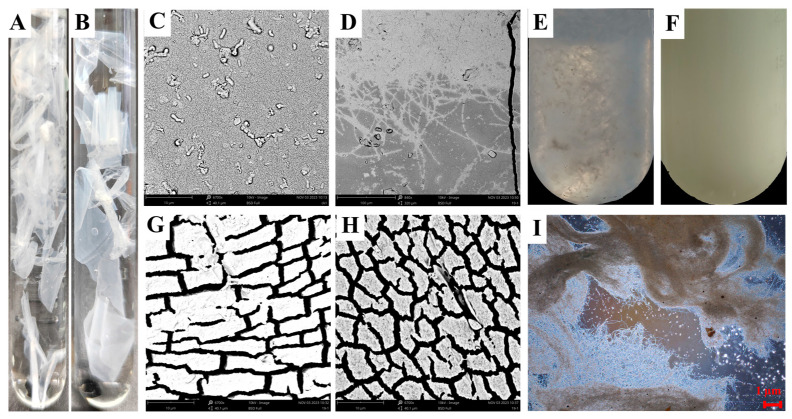
Degradation effect of *T. harzianum* T4 on PBAT films and powder. (**A**) PBAT films in inorganic salt medium after 5-day co-culture with *T. harzianum* T4. (**B**) PBAT films in inorganic salt medium after 5-day culture without *T. harzianum* T4. (**C**) SEM observation of PBAT film surface without *T. harzianum* T4. (**D**) SEM observation of the mycelia on PBAT film surface after 5-day co-culture. (**E**) The state of the inorganic salt medium after *T. harzianum* T4 utilization of PBAT powder. (**F**) The state of inorganic salt medium without *T. harzianum* T4. (**G**,**H**) Surface cracking of PBAT film after interaction with *T. harzianum* T4. (**I**) Microscopic view of *T. harzianum* T4 hyphal proliferation on PBAT powder.

When *T. harzianum* T4 was incubated with PBAT substrate films in inorganic salt medium for 5 days, significant film degradation was observed (Figure 2A,B). Macroscopically, the film surface in contact with mycelia developed visible cracks and roughness after 5 days, in stark contrast to the smooth surface of the control group (Figure 2C,G,H). Scanning electron microscopy (SEM) revealed that *T. harzianum* T4 mycelia penetrated and colonized the film matrix, forming numerous cavities and voids (Figure 2D). The film fragmentation prevented testing of mechanical properties (e.g., tensile strength and elongation at break), indicating severe structural degradation of PBAT films under *T. harzianum* T4 treatment. When *T. harzianum* T4 was cultured with PBAT powder in inorganic salt medium, the fungus utilized PBAT as a carbon source for reproduction, leading to hyphal proliferation and clarification of the culture medium (Figure 2E,I), whereas the *T. harzianum* T4-free control medium with PBAT powder remained turbid (Figure 2F).

### 2.3. The Effects of T. harzianum T4 and Microplastics on Plant Agronomic Traits

**Figure 3:**
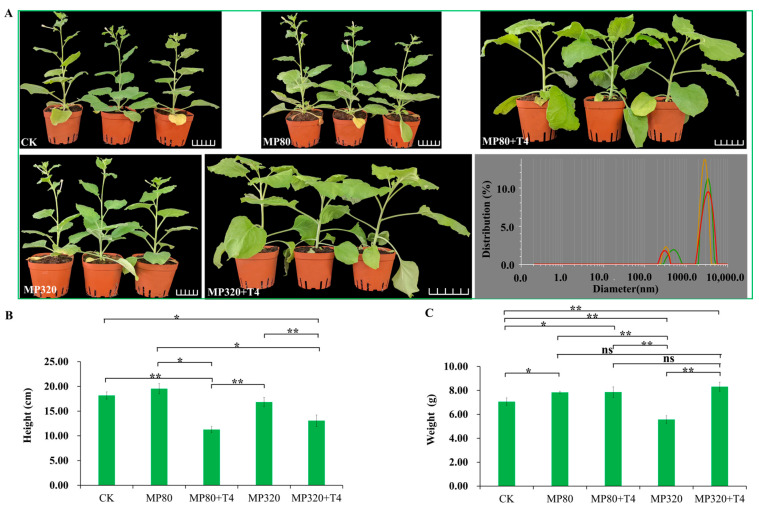
Effect of the aged microplastics and *T. harzianum* T4 on *N. benthamiana* agronomic trait. (**A**) Phenotypic comparison of *N. benthamiana* under different treatment conditions and particle sizes of PBAT powder. (**B**) Plant height statistics of *N. benthamiana* across various treatments. (**C**) Weight statistics across various treatments. The yellow, red, and green lines in (**A**) correspond to the results of three replicate measurements. Values indicate the *p*-value (significant at *p*-value < 0.05) of the results of pairwise comparison using ANOVA (*: adjust *p*-value < 0.05; **: adjust *p*-value < 0.01; ns: no significant difference. This is the same below).

In this study exploring the impacts of *T. harzianum* T4 and aged microplastics on the agronomic traits of *N. benthamiana*, a series of intriguing findings emerged. When 320 mg/kg of aged microplastics (MP320) was applied alone, it significantly hindered the plant growth of *N. benthamiana* (Figure 3A). The height and weight were decreased by 7.33% and 21.17%, respectively, with a significant weight difference compared to the control (CK) (Figure 3B,C). However, supplementing MP320 with *T. harzianum* T4 (MP320+T4) shifted this trend, while height still declined (from 16.83 cm to 13.07 cm), weight significantly increased by 49.34% (from 5.56 g to 8.31 g) (Figure 3B,C). Relative to the CK, the combined MP320+T4 treatment boosted plant weight by 17.72%. For the 80 mg/kg aged microplastic treatment (MP80), plant height and weight increased by 7.52% and 11.01% compared to the CK, respectively (Figure 3B,C). When *T. harzianum* T4 was added (MP80+T4), growth trends resembled MP320+T4. However, there was a significant height difference between MP80+T4 (19.53 cm) and MP80 (11.27 cm), but no significant differences in weight (7.84 g vs. 7.85 g), and the weight of MP80+T4 was slightly higher than that of MP80 (Figure 3B,C). Collectively, these results demonstrate that high concentrations of aged microplastics significantly and negatively impact plant agronomic traits, while *T. harzianum* T4 effectively mitigates microplastic stress, particularly at higher microplastic concentrations.

### 2.4. Effects of T. harzianum and Aged Microplastics on Chlorophyll, MDA, and O_2_^−^

**Figure 4:**
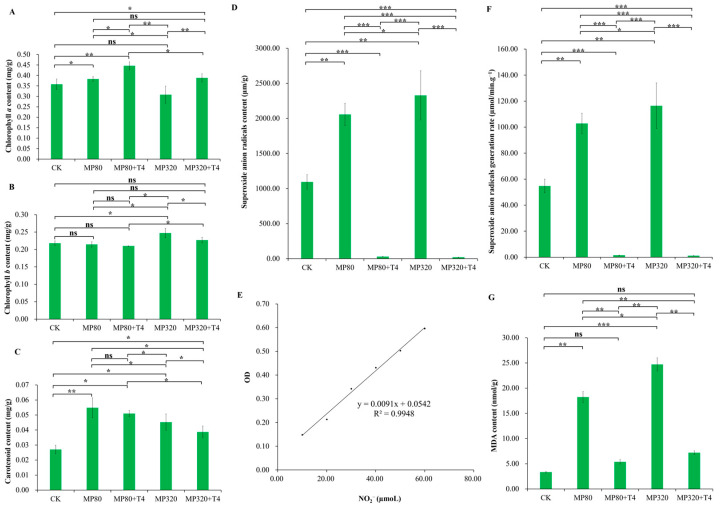
Effect of the aged microplastics and *T. harzianum* T4 on chlorophyll, MDA, and superoxide anion radicals. (**A**) Chlorophyll *a* content in *N. benthamiana* under different treatments. (**B**) Chlorophyll *b* content under different treatments. (**C**) Carotenoid content under different treatments. (**D**) Superoxide anion radical content under different treatments. (**E**) Standard curve and equation for calculating superoxide anion radical concentration. (**F**) Superoxide anion radical production rate under different treatments. (**G**) MDA content under different treatments. Values indicate the *p*-value (significant at *p*-value < 0.05) of the results of pairwise comparison using ANOVA (*: adjust *p*-value < 0.05; **: adjust *p*-value < 0.01; ***: adjust *p*-value < 0.001; ns: no significant difference. This is the same below).

Notably, when examining the impacts of *T. harzianum* T4 and aged microplastics on chlorophyll, MDA, and ROS in *N. benthamiana*, distinct patterns emerged. When *N. benthamiana* was cultivated with aged microplastics, chlorophyll *a* (C*_a_*) content showed contrasting trends between the two microplastic concentrations: MP80 (0.38 mg/g) > CK (0.36 mg/g) > MP320 (0.31 mg/g) (Figure 4A). This indicates low concentrations of aged microplastics slightly elevated C*_a_* levels, while high concentrations significantly reduced C*_a_* levels, suggesting a dose-dependent inhibition of photosynthesis. When *T. harzianum* T4 was co-applied (MP80+T4 and MP320+T4), C*_a_* content increased by 16.51% (MP80+T4 vs. MP80) and 26.08% (MP320+T4 vs. MP320), with both treatments exceeding CK levels (Figure 4A). Notably, C*_b_* in MP80 (0.21 mg/g) was 1.67% lower than the CK, while MP320 (0.25 mg/g) showed a 13.35% increase relative to the CK (Figure 4B). An intriguing observation was the decrease in chlorophyll-*b* content when *T. harzianum* T4 was added to the aged microplastic treatments. The content was as follows: MP320 (0.25 mg/g) > MP320+T4 (0.23 mg/g) > CK (0.22 mg/g) > MP80 (0.21 mg/g) > MP80+T4 (0.20 mg/g) (Figure 4B). In terms of carotenoids, compared to the CK, exogenous aged microplastics (with or without *T. harzianum* T4) generally increased carotenoid content (Figure 4C). However, similar to chlorophyll-*b*, *T. harzianum* T4 reduced carotenoid levels in aged microplastic-treated groups. The order was as follows: MP80 (0.055 mg/g) > MP80+T4 (0.051 mg/g) > MP320 (0.045 mg/g) > MP320+T4 (0.039 mg/g) > CK (0.027 mg/g) (Figure 4C).

Compared with the control group (CK = 1094.85 µm/g), the content of superoxide anion radicals (O_2_^−^) in the aged microplastic-treated groups (MP80 = 2056.37 µm/g and MP320 = 2328.58 µm/g) significantly increased, showing 1.88-fold and 2.13-fold changes, respectively (Figure 4D,F). This indicates that aged microplastic treatment can elevate the content of superoxide anion radicals and induce oxidative stress. When compared with the corresponding aged microplastic-treated groups (MP80 and MP320), the MP80+T4 and MP320+T4 groups exhibited a significant reduction in superoxide anion free radical content, to only 39.48% and 32.89% of the respective microplastic-treated groups (MP80+T4 = 811.89 µm/g and MP320+T4 = 765.87 µm/g), and the superoxide anion generation rate was also significantly decreased (Figure 4D,F). This suggests that the addition of *T. harzianum* T4 can notably mitigate the accumulation of superoxide anion free radicals induced by microplastics and relieve oxidative stress.

The MDA content in the MP80 (18.22 nmol/g) and MP320 (24.71 nmol/g) groups was significantly higher than that in the control group (CK = 3.36 nmol/g), demonstrating that aged microplastic treatment exacerbated membrane lipid peroxidation in *N. benthamiana* (Figure 4G). As a product of membrane lipid peroxidation, MDA content increased by 5.43-fold in MP80 and 7.36-fold in MP320 relative to the CK (Figure 4G). The MDA content in the MP80+T4 group (5.39 nmol/g) was significantly reduced to 29.57% of that in MP80, and the MP320+T4 group (7.20 nmol/g) showed a similar trend, decreasing to 29.15% of MP320 (Figure 4G). These results indicate that *T. harzianum* T4 treatment effectively mitigates membrane lipid peroxidation induced by aged microplastics, thereby protecting cell membrane structure and function. By reducing the accumulation of superoxide anion radicals and MDA, *T. harzianum* T4 alleviates oxidative stress caused by aged microplastics.

### 2.5. Effects of T. harzianum and Microplastics on Enzyme Activities

**Figure 5:**
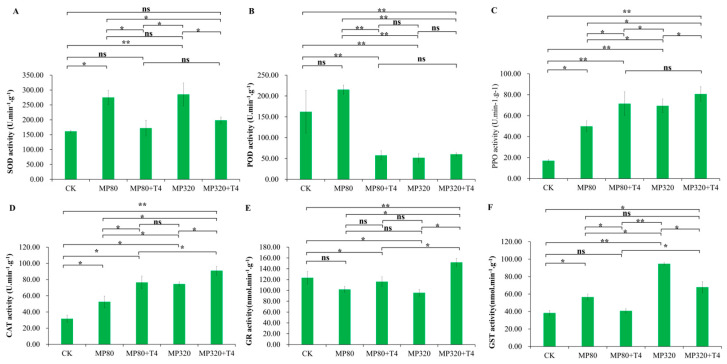
Effect of the aged microplastics and *T. harzianum* T4 on enzyme activities (**A**) SOD activity in *N. benthamiana* under different treatments; (**B**) POD activity under different treatments; (**C**) PPO Activity under different treatments; (**D**) CAT activity under different treatments; (**E**) GR Activity under different treatments; (**F**) GST activity under different treatments. Values indicate the *p*-value (significant at *p*-value < 0.05) of the results of pairwise comparison using ANOVA (*: adjust *p*-value < 0.05; **: adjust *p*-value < 0.01; ns: no significant difference. This is the same below).

Low and high concentrations of microplastics (MP80 and MP320) both induced an increase in SOD activity, with MP80 (275.18 U·min^−1^·g^−1^) and MP320 (285.52 U·min^−1^·g^−1^) showing 1.7-fold and 1.77-fold increases compared to the control (CK, 161.40 U·min^−1^·g^−1^), respectively (Figure 5A). When *T. harzianum* T4 was combined with aged microplastics, SOD activity in the low-concentration group (MP80+T4) dropped significantly to 62.58% of the MP80 group (Figure 5A). In the high-concentration group (MP320+T4), SOD activity significantly decreased by 30.53% compared to MP320, though it was 22.89% higher than the CK, which does not show significant difference (Figure 5A). These results suggest that *T. harzianum* T4 alleviates the microplastic stress-induced upregulation of SOD activity.

Aged microplastics induced concentration-dependent POD activity responses: a slight increase at low concentrations (MP80, 215.36 U·min^−1^·g^−1^) and a significant decrease at high concentrations (MP320, 51.71 U·min^−1^·g^−1^), with values ordered as MP80 > CK (162.26 U·min^−1^·g^−1^) > MP320 (Figure 5B). In combined treatments with *T. harzianum* T4, low-concentration MP80+T4 showed a sharp POD activity reduction (26.71% below MP80 and 35.45% below the CK), while high-concentration MP320+T4 (60.24 U·min^−1^·g^−1^) remained lower than the CK but was 1.16-fold higher than MP320 (Figure 5B). Among all treatment groups, MP80 alone exhibited the highest POD activity, indicating a complex regulatory pattern of POD under microplastic stress and biological mitigation.

When *N. benthamiana* was cultivated with aged microplastics, polyphenol oxidase (PPO) activity increased at both low (MP80, 49.87 U·min^−1^·g^−1^) and high (MP320, 69.43 U·min^−1^·g^−1^) concentrations of microplastics compared to the control (CK), with MP320 showing a more pronounced induction (Figure 5C). This indicates that aged microplastics can induce PPO activity in plants as a defense response to microplastic stress. Co-treatment with *T. harzianum* T4 further enhanced PPO activity in a concentration-dependent manner: at low microplastic concentrations with *T. harzianum* T4, MP80+T4 (71.47 U·min^−1^·g^−1^) exhibited a 1.43-fold increase over MP80 and a 4.21-fold increase over the CK; at high concentrations with *T. harzianum* T4, MP320+T4 (80.63 U·min^−1^·g^−1^) showed a more significant rise, reaching 4.75-fold over the CK and 1.16-fold over MP320 (Figure 5C). These findings suggest a synergistic effect between the defense mechanisms of *T. harzianum* T4 and microplastic-induced stress, whereby *T. harzianum* T4 promotes PPO production to enhance plant stress tolerance.

Exposure to aged microplastics significantly increased CAT activity compared to the control group (CK), with a clear concentration-dependent trend. CAT activity followed the order: MP320 (74.47 U·min^−1^·g^−1^) > MP80 (52.54 U·min^−1^·g^−1^) > CK (31.55 U·min^−1^·g^−1^) (Figure 5D). When *T. harzianum* T4 was co-applied with aged microplastics, CAT activity further increased in both MP80+T4 (76.51 U·min^−1^·g^−1^) and MP320+T4 (90.94 U·min^−1^·g^−1^) groups (Figure 5D). Specifically, MP80+T4 showed a 1.46-fold increase over MP80, while MP320+T4 exhibited a 1.22-fold increase over MP320, maintaining the same concentration-dependent pattern as the microplastic-only treatments (Figure 5D). These results highlight the coordinated antioxidant defense mechanism of *T. harzianum* T4 in enhancing CAT activity under microplastic stress.

When *N. benthamiana* was treated with aged microplastics, glutathione reductase (GR) activity showed concentration-dependent inhibition (Figure 5E), with both low (MP80, 101.84 nmol·min^−1^·g^−1^) and high (MP320, 95.59 nmol·min^−1^·g^−1^) concentrations yielding lower activity than the blank control (CK, 123.28 nmol·min^−1^·g^−1^). This suggests that aged microplastics directly suppress GR-mediated glutathione recycling. However, co-treatment with *T. harzianum* T4 significantly alleviated this inhibition: GR activity in MP80+T4 (116.13 nmol·min^−1^·g^−1^) and MP320+T4 (151.87 nmol·min^−1^·g^−1^) increased by 14.04% and 58.88% compared to microplastic-only groups, respectively, indicating restored redox homeostasis via enhanced glutathione regeneration (Figure 5E).

In contrast, glutathione S-transferase (GST) displayed opposing trends: aged microplastics induced significant GST activity elevation, particularly at high concentrations (MP320, 94.71 nmol·min^−1^·g^−1^), which was 146.91% higher than the CK (38.36 nmol·min^−1^·g^−1^) (Figure 5F). However, *T. harzianum* T4 co-treatment attenuated this induction: GST activity in MP80+T4 (40.72 nmol·min^−1^·g^−1^) and MP320+T4 (67.87 nmol·min^−1^·g^−1^) decreased by 28% and 28.33% relative to MP80 (56.56 nmol. min^−1^ g^−1^) and MP320, respectively, despite remaining 6.16% and 76.94% higher than the CK (Figure 5F). This suggests that the combined stressor disrupts GST-dependent detoxification pathways, potentially leading to toxic compound accumulation via reduced conjugative detoxification efficiency.

### 2.6. Effects of T. harzianum and Microplastics on N. benthamiana Gene Expression

**Figure 6:**
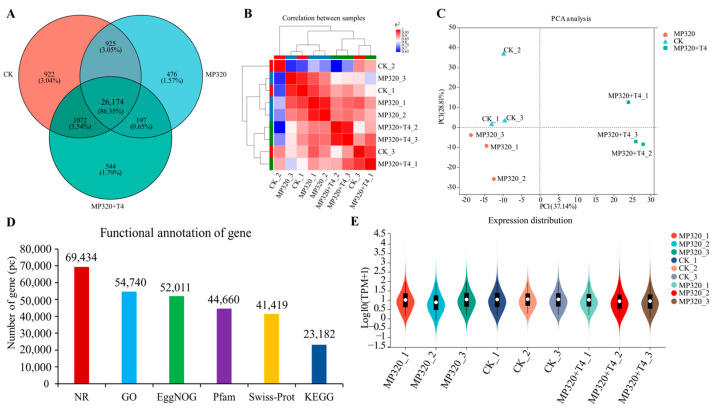
Multidimensional comprehensive analysis of transcriptome data. (**A**) Venn diagram of annotated gene overlap between samples. (**B**) Heatmap of correlation between different samples. (**C**) PCA analysis of transcriptome data. (**D**) Statistical analysis of gene function annotation. (**E**) Violin plot of gene expression distribution.

In this study, RNA-seq was utilized to systematically explore the impact of high-concentration aged microplastics and *T. harzianum* T4 on gene expression in *N. benthamiana*. Following rigorous quality control and data filtering, the RNA-seq analysis generated 40.43–43.90 million clean reads (Q30 base ratio: 94.03–94.22%) from the CK library, 44.23–46.69 million reads (Q30: 94.03–94.18%) from the MP320 library, and 39.39–47.43 million reads (Q30: 94.05–94.08%) from the MP320+T4 library (Supplementary Table S1). Mapping to the *N. benthamiana* reference genome (Niben261; https://solgenomics.net, accessed on 18 October 2024) yielded 39.71–43.10 million, 41.50–45.74 million, and 38.71–46.53 million aligned reads for the CK, MP320, and MP320+T4, respectively, with mapping rates of 98.17–98.23%, 97.97–98.16%, and 97.81–98.27% (Supplementary Table S1). A total of 29,093, 27,772, and 27,987 genes were identified in the CK, MP320, and MP320+T4 groups (Supplementary Table S2 and Figure 6A). The high biological replicate correlation (R^2^ > 0.99) validated the reliability of the transcriptomic data (Figure 6B).

In the PCA analysis, PC1 (37.14%) and PC2 (28.81%) collectively explained 65.95% of the total variance. MP320 samples clustered distinctly on the left of the PC1 axis, forming a clear separation from the CK samples on the right, highlighting fundamental differences in gene expression profiles between the two groups. MP320+T4 samples were distributed between the two clusters with partial overlap with the CK, suggesting that *T. harzianum* T4 may partially reverse MP320-induced expression changes while retaining a unique pattern. Tight intragroup clustering of samples reflected the high consistency of biological replicates. These results indicate that the core effect of MP320 treatment is captured along PC1, whereas the regulatory effect of *T. harzianum* T4 primarily acts by modulating gene expression along PC2 (Figure 6C). Genes were functionally annotated using EggNOG, PFAM, KEGG, Swissprot, GO, and NR databases (Figure 6D). Annotation counts varied across databases: the NR database annotated the most genes (69,434), while KEGG annotated the fewest (23,182), reflecting differing coverage of gene functional annotations. A violin plot revealed distinct patterns of gene expression distribution across samples, with some (e.g., MP320 and the CK) showing varying degrees of dispersion, indicating differences in expression central tendency and spread between groups (Figure 6E).

**Figure 7:**
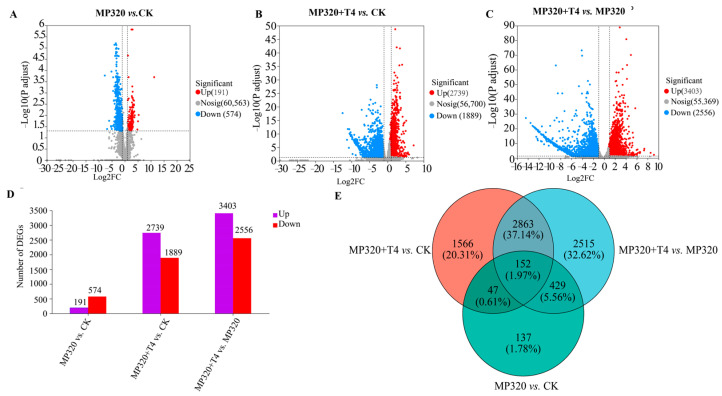
Analysis of DEGs in the transcriptome between different sample combinations. (**A**) Volcano of DEGs between MP320 and CK groups. (**B**) Volcano of DEGs between MP320+T4 and CK groups. (**C**) Volcano of DEGs between MP320+T4 and MP320 groups. (**D**) Statistical analysis of the number of DEGs between different sample combinations. (**E**) Venn of DEGs between different sample combinations.

Using |log_2_FC| > 2 and FDR < 0.05 as filtering criteria, a total of 7710 differentially expressed genes (DEGs) were identified in *N. benthamiana* across the CK, MP320, and MP320+T4 groups (Supplementary Tables S3–S5). Specifically, the comparisons MP320 vs. CK, MP320+T4 vs. CK, and MP320+T4 vs. MP320 yielded 765, 4628, and 5959 DEGs, respectively. Among these, 191, 2739, and 3403 DEGs were upregulated, while 574, 1889, and 2556 were downregulated in the three comparison groups (Supplementary Tables S3–S5, Figure 7A–D). A Venn diagram analysis revealed 152 common DEGs across all three comparisons (Figure 7E).

**Figure 8:**
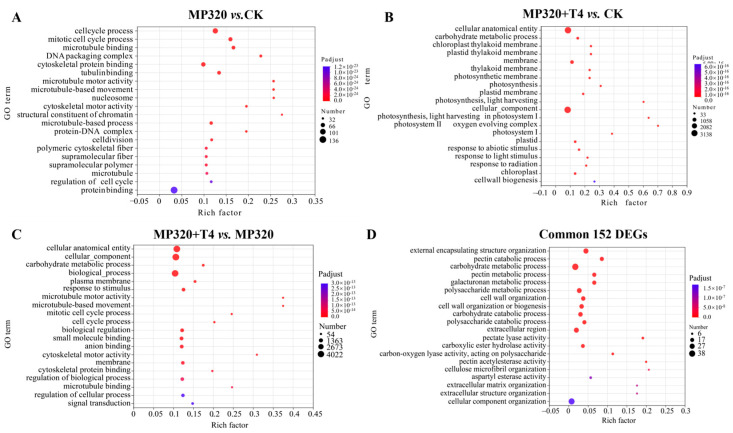
GO enrichment analysis of differentially expressed genes. (**A**) Bubble plot of GO enrichment analysis of DEGs in MP320 vs. CK. (**B**) Bubble plot of GO enrichment analysis of DEGs in MP320+T4 vs. CK. (**C**) Bubble plot of GO enrichment analysis of DEGs in MP320+T4 vs. MP320. (**D**) Bubble plot of GO enrichment analysis of 152 DEGs shared among the three comparison groups.

Gene Ontology (GO) analysis was conducted to explore the roles of genes involved in *N. benthamiana* responses to aged microplastics and *T. harzianum* T4 across the biological process (BP), cellular component (CC), and molecular function (MF) categories. Aged microplastic exposure (MP320 vs. CK) induced significant enrichment of 765 DEGs in cell cycle regulation (e.g., cell cycle process, *p* = 2.90 × 10^−58^; mitotic cell cycle, *p* = 1.47 × 10^−46^) and cytoskeletal functions (e.g., microtubule binding, *p* = 4.67 × 10^−42^). Co-treatment with *T. harzianum* T4 (MP320+T4 vs. CK) led to 4628 DEGs enriched in carbohydrate metabolism (*p* = 3.11 × 10^−33^) and chloroplast thylakoid membranes (*p* = 6.22 × 10^−33^), while MP320+T4 vs. MP320 (5959 DEGs) highlighted cellular organization (*p* = 9.33 × 10^−45^) and plasma membrane components (*p* = 6.15 × 10^−21^) (Figure 8A–C). The 152 common DEGs across all comparisons were primarily associated with cell wall metabolism (e.g., pectin catabolic process, *p* = 1.69 × 10^−19^) and carbohydrate metabolic pathways (*p* = 5.25 × 10^−19^) (Figure 8D). Collectively, aged microplastics predominantly altered BP/MF terms related to cell cycle and cytoskeletal dynamics, whereas *T. harzianum* T4 co-treatment modulated processes such as carbohydrate metabolism and stimulus responses, reflecting distinct regulatory patterns of single and combined stressors.

**Figure 9:**
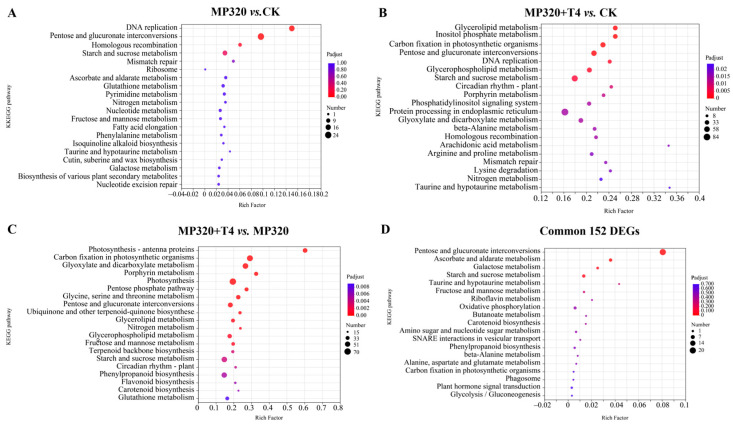
KEGG enrichment analysis of DEGs under different treatment combinations. (**A**) KEGG enrichment analysis of DEGs in MP320 vs. CK. (**B**) KEGG enrichment analysis of DEGs in MP320+T4 vs. CK. (**C**) KEGG enrichment analysis of DEGs in MP320+T4 vs. MP320. (**D**) KEGG enrichment analysis of 152 Shared DEGs.

KEGG pathway analysis (*p* < 0.05) revealed that DEGs from all comparisons groups were significantly enriched in 40 metabolic processes. In MP320 vs. CK, enrichment was observed in DNA replication (*p* = 3.70 × 10^−10^) and pentose/glucuronate interconversions (*p* = 8.71 × 10^−10^). MP320+T4 vs. MP320 highlighted inositol phosphate metabolism (*p* = 1.21 × 10^−4^), carbon fixation in photosynthetic organisms (*p* = 1.34 × 10^−4^), and glycerolipid metabolism (*p* = 1.83 × 10^−4^), while MP320+T4 vs. CK showed strong enrichment in photosynthesis-antenna proteins (*p* = 2.76 × 10^−23^), carbon fixation (*p* = 1.52 × 10^−14^), and glyoxylate/dicarboxylate metabolism (*p* = 1.13 × 10^−11^) (Figure 9A–C). Notably, aged microplastics and *T. harzianum* T4 co-treatment both impacted DNA replication and pentose/glucuronate interconversions, as exemplified by genes like Niben261Chr02g0822017 (log_2_FC = −11.30 in MP320+T4 vs. MP320, 2.32 in MP320 vs. CK, −8.96 in MP320+T4 vs. CK) and Niben261Chr02g0958006 (log_2_FC = −12.11, 2.52, −9.57 across the same comparisons). In DNA replication pathways, microplastic-suppressed genes (e.g., Niben261Chr01g1441013, Niben261Chr07g1000008) were upregulated by *T. harzianum* T4 co-treatment, potentially linking to enhanced leaf expansion and plant biomass. For the 152 common DEGs, KEGG analysis identified enrichment in pentose/glucuronate interconversions, ascorbate/aldarate metabolism, starch/sucrose metabolism, and galactose metabolism (Figure 9D). Key genes included Niben261Chr08g1038012 (log_2_FC = −7.22, −10.02, 2.77) and Niben261Chr02g0822017 (log_2_FC = −8.96, −11.30, 2.32) across comparisons. qRT-PCR validation of nine common DEGs (including *WRKY40*, *WRKY40A*, and *WRKY70* transcription factors) confirmed consistency with RNA-seq data, supporting transcriptome reliability (Supplementary Figure S1). Based on functional annotation, fold changes, and agronomic trait correlations, *WRK40* (Niben261Chr08g0154013), *WRK40A* (Niben261Chr18g1442002), and *CYPR4* (Niben261Chr10g0687008) were prioritized as candidate genes for functional characterization.

### 2.7. Metagenomic Data Description

This study performed metagenomic sequencing on nine samples (CK, MP320, MP320+T4; *n* = 3 per group), generating 117.9 Gb of high-quality clean data with an average depth of 13.1 Gb/sample (Supplementary Table S6). After assembly, gene prediction, and filtering of sequences < 100 bp, 1,396,407 (MP320), 1,338,769 (CK), and 1,425,188 (MP320+T4) genes were identified. Clustering at 90% identity and coverage yielded 3,920,991 non-redundant genes (total length: 1.97 × 10^9^ bp; average length: 502.11 bp). Using a similarity threshold of ≥95% for abundance calculation, 2,514,763 (MP320), 2,496,018 (CK), and 2,553,272 (MP320+T4) genes were quantified (Supplementary Table S6). Notably, the MP320+T4 microbiome harbored the highest gene richness (2,553,272 genes), exceeding both the MP320 (2,514,763) and CK (2,496,018) groups (Supplementary Table S6).

**Figure 10:**
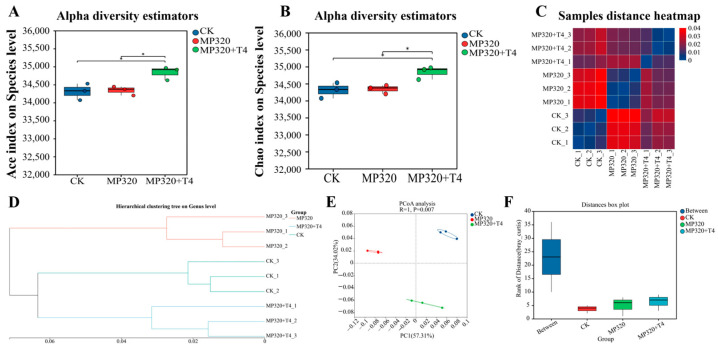
Diversity and difference analysis of microbial communities in different treatment groups. (**A**): Ace index alpha diversity comparison; (**B**): Chao index alpha diversity comparison; (**C**): sample distance heatmap; (**D**): genus horizontal clustering tree; (**E**): PCoA analysis chart; (**F**): intergroup distance boxplot. Values indicate the *p*-value (significant at *p*-value < 0.05) of the results of pairwise comparison using ANOVA (*: adjust *p*-value < 0.05. This is the same below).

Alpha diversity analysis, which assesses microbial community richness and diversity via indices like Ace and Chao, revealed significant differences in Ace and Chao values between the MP320+T4 group and the other two treatments (MP320 and CK) (Figure 10A,B). Hierarchical clustering analysis showed distinct clustering of the three treatments, aged microplastics, control, and microplastics combined with *T. harzianum* T4, with significant inter-sample distance (Figure 10C,D). Principal coordinate analysis (PCoA) based on the NR database demonstrated separation of the three groups, with PC1 explaining 57.31% of the variance (Figure 10E), indicating significant differences in overall sample composition. Analysis of similarity (ANOSIM), a nonparametric test for comparing between-group versus within-group differences, confirmed the highest rank of between-group distances (Figure 10F), validating the biological significance of the sample grouping.

### 2.8. Classification of the Microbiome

**Figure 11:**
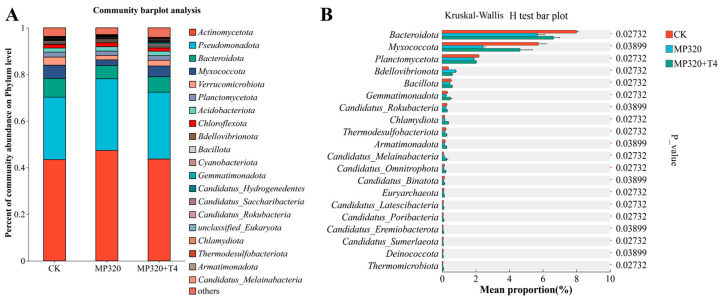
Analysis of microbial community composition and differences in phylum level among different treatment groups. (**A**) Microbial community phylum level composition in different treatment groups. (**B**) Differences in microbial community phylum levels among different treatment groups by the Kruskal–Wallis H Test.

Non-redundant gene sets were annotated against the NR database to characterize species composition. At the phylum level, *Actinomycetota* (49.47%), *Pseudomonadota* (25.29%), *Bacteroidota* (6.42%), and *Myxococcota* (5.22%) dominated across all three treatment groups. Specifically, the control group (CK) showed *Actinomycetota* (43.42%) and *Pseudomonadota* (26.82%), while MP320 significantly increased *Actinomycetota* (47.36%) and *Pseudomonadota* (30.76%). In contrast, MP320+T4 reduced these phyla to 43.68% and 28.69%, respectively, while increasing *Bacteroidota* (*p* = 0.027), *Myxococcota* (*p* = 0.039), and *Planctomycetota* (*p* = 0.027) (Figure 11A; Supplementary Table S7). Aged microplastics alone induced significant shifts: *Actinomycetota* and *Pseudomonadota* abundances rose, whereas *Bacteroidota* dropped sharply (Figure 11B). *T. harzianum* T4 co-treatment further modulated the microbiota, notably elevating *Bacteroidota* and *Myxococcota* and decreasing *Bdellovibrionota* relative to MP320 (Figure 11A). These results indicate that *T. harzianum* T4 alters the structure and diversity of *N. benthamiana* soil microbial communities.

**Figure 12:**
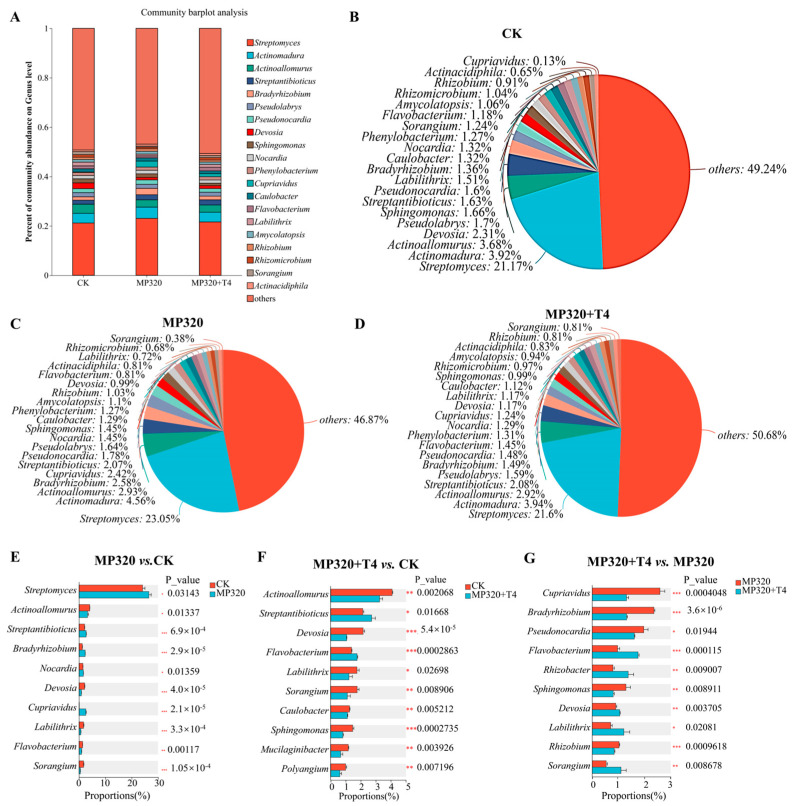
Composition and intergroup differences at the genus level among different treatment groups. (**A**) Composition of genera in different treatment groups. (**B**) Composition of genus level in the CK. (**C**) Composition of genus level in MP320. (**D**) Composition of genus level in MP320+T4. (**E**) Differences at the genus level in MP320 vs. CK. (**F**) Differences at the genus level in MP320+T4 vs. CK. (**G**) Differences at the genus level in MP320+T4 vs. MP320. Values indicate the *p*-value (significant at *p*-value < 0.05) of the results of pairwise comparison using ANOVA (*: adjust *p*-value < 0.05; **: adjust *p*-value < 0.01; ***: adjust *p*-value < 0.001. This is the same below).

At the genus level, the control group was dominated by *Streptomyces* (21.17%), *Actinomadura* (3.92%), and *Actinoallomurus* (3.68%). These genera remained dominant in both aged microplastic (MP320) and MP320+T4 treatments (Figure 12A–D). However, aged microplastics alone significantly increased *Streptomyces*, *Streptantibioticus*, and *Cupriavidus* abundances while reducing *Actinoallomurus* and *Devosia* (Figure 12C–E). In the MP320+T4 group, *Streptomyces*, *Actinomadura*, and *Actinoallomurus* remained predominant (Figure 12D–F). The statistical analysis revealed distinct shifts, in which *Sphingomonas*, *Pseudonocardia*, *Bradyrhizobium*, and *Cupriavidus* were significantly reduced, whereas *Labilithrix*, *Rhizobacter*, and *Flavobacterium* showed increased abundance (Figure 12G).

### 2.9. Diversity, Abundance, and Classes of the Drug Resistance and Carbohydrate-Active Enzymes

**Figure 13:**
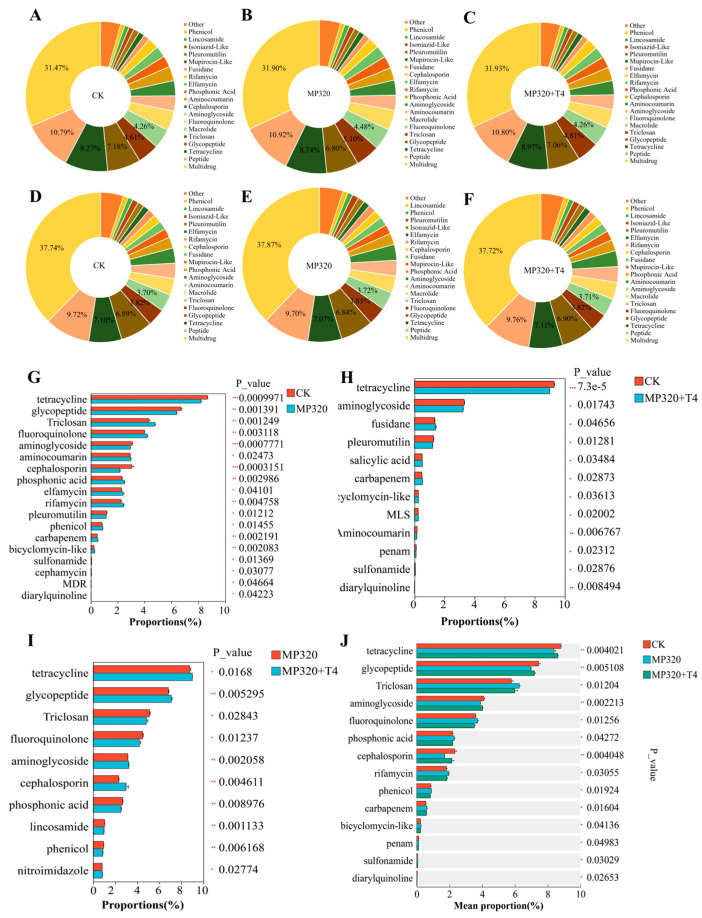
Composition and differential analysis of antibiotic resistance genes in different treatment groups. (**A**) Abundance composition of ARGs in CK group. (**B**) Abundance composition of ARGs in MP320. (**C**) Abundance composition of ARGs in MP320+T4. (**D**) Count composition of ARGs in CK. (**E**) Count composition of ARGs in MP320. (**F**) Count composition of ARGs in MP320+T4. (**G**) Abundance differences of ARGs in MP320 vs. CK. (**H**) Abundance differences of ARGs in MP320+T4 vs. CK. (**I**) Abundance differences of ARGs in MP320+T4 vs. MP320. (**J**) Comparative analysis of the average proportion of ARGs in the three groups. Values indicate the *p*-value (significant at *p*-value < 0.05) of the results of pairwise comparison using ANOVA (*: adjust *p*-value < 0.05; **: adjust *p*-value < 0.01; ***: adjust *p*-value < 0.001. This is the same below).

To characterize antibiotic resistance genes (ARGs) in soil metagenomes across three treatments, 1359 ARGs were identified via CARD database comparison and categorized into 36 resistance classes. Multidrug resistance dominated (40.72% of total ARGs), followed by peptide, tetracycline, glycopeptide, and triclosan resistance, which collectively accounted for 31.85% (CK), 31.56% (MP320), and 31.64% (MP320+T4) of total ARGs (Figure 13A–C). Multidrug resistance ARG abundance was significantly higher in aged microplastic and *T. harzianum* T4-treated soils than in the CK (Figure 13A–C). Among 36 classes, multidrug resistance harbored the most unique ARGs (553, e.g., *gyrA*, efflux pumps, *bla* families), followed by aminoglycoside (94, e.g., *AAC (3)*, *AAC (6′)*), glycopeptide (80, e.g., *vanS*, *vanR*), peptide (74), and tetracycline (75) (Supplementary Table S8; Figure 13D–F). Identified resistance mechanisms included reduced permeability, target alteration/replacement/protection, efflux, and inactivation (Supplementary Table S8). Core ARGs (occurrence ≥ 90%, abundance > median) numbered 568 (CK), 577 (MP320), and 582 (MP320+T4), primarily associated with multidrug, tetracycline, glycopeptide, and peptide resistance. Statistical analyses revealed 18 significantly different classes in MP320 vs. CK (e.g., *tetA5*, *van*, *fabG*; Figure 13G), 12 in MP320+T4 vs. CK (e.g., *tetA*, *novA*, *fusA*; Figure 13H), and 10 in MP320+T4 vs. MP320 (e.g., *tetA58*, *vanR*, *fabG*; Figure 13I). Crossgroup comparisons identified 14 significantly different classes, with tetracycline (*p* = 0.004), glycopeptide (*p* = 0.005), and triclosan (*p* = 0.004) being most abundant (Figure 13J).

**Figure 14:**
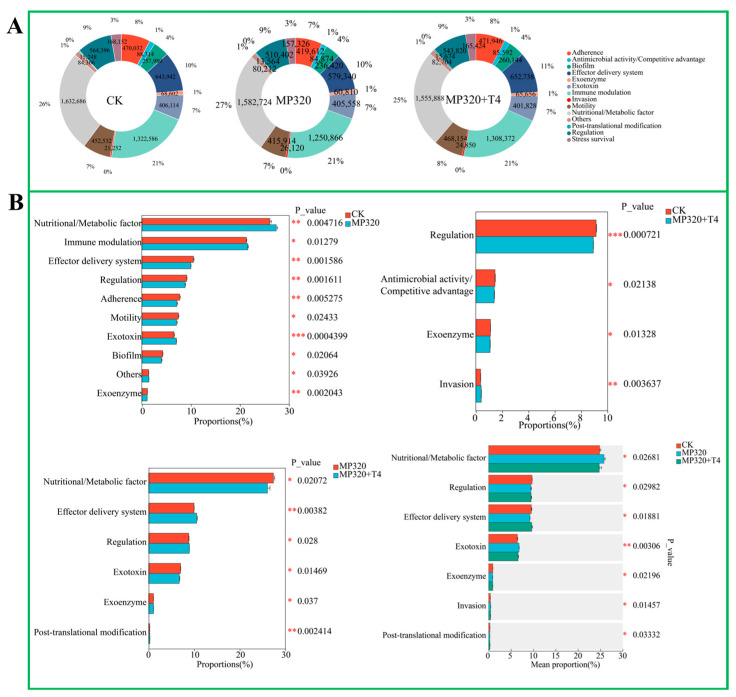
Composition and differential analysis of VFs in different treatment groups. (**A**) Abundance composition of VFs in the three groups. (**B**) Abundance differences of VFs in MP320 vs. CK, MP320+T4 vs. CK, MP320+T4 vs. MP320, and comparative analysis of the average proportion of VFs. Values indicate the *p*-value (significant at *p*-value < 0.05) of the results of pairwise comparison using ANOVA (*: adjust *p*-value < 0.05; **: adjust *p*-value < 0.01; ***: adjust *p*-value < 0.001. This is the same below).

In the analysis of virulence factors (VFs) in soil metagenomes across three treatments, 1032 VFs were annotated and categorized into 13 functional classes, including immune modulation, effector delivery systems, nutritional/metabolic factors, adherence, stress survival, antimicrobial activity/competitive advantage, biofilm formation, regulation, exoenzymes, exotoxins, invasion, motility, post-translational modification, and others. The CK group exhibited a baseline distribution of VF functions, with nutritional/metabolic factors accounting for 26%; immune modulation, 21%; and adherence, 8% (Supplementary Table S9; Figure 14A). Aged microplastic treatment (MP320) slightly altered this composition, increasing nutritional/metabolic factors to 27%, while decreasing adherence to 7%, indicating stress-induced shifts in virulence profiles. Co-treatment with *T. harzianum* T4 (MP320+T4) further rebalanced VF distributions: nutritional/metabolic factors decreased to 25% compared to the CK, while effector delivery systems and motility each increased by 1% (Supplementary Table S9; Figure 14A). Statistical analyses revealed significant intergroup differences: MP320 vs. CK showed altered proportions of nutritional/metabolic factors (*p* = 0.004716) and immune modulation (*p* = 0.01279), whereas MP320+T4 vs. MP320 highlighted shifts in nutritional/metabolic factors (*p* = 0.0272) and effector delivery systems (*p* = 0.00382), indicating *T. harzianum* T4-mediated reversal of microplastic-induced VF changes (Figure 14B). Crossgroup comparisons identified significant differences in regulatory functions and antimicrobial/competitive advantage activities (e.g., regulation *p* = 0.000721 for MP320+T4 vs. CK), reflecting complex adjustments to the microbial virulence network under combined stress (Figure 14B). Collectively, these findings demonstrate that *T. harzianum* T4 modulates the impact of aged microplastics on soil microbial virulence factor profiles.

**Figure 15:**
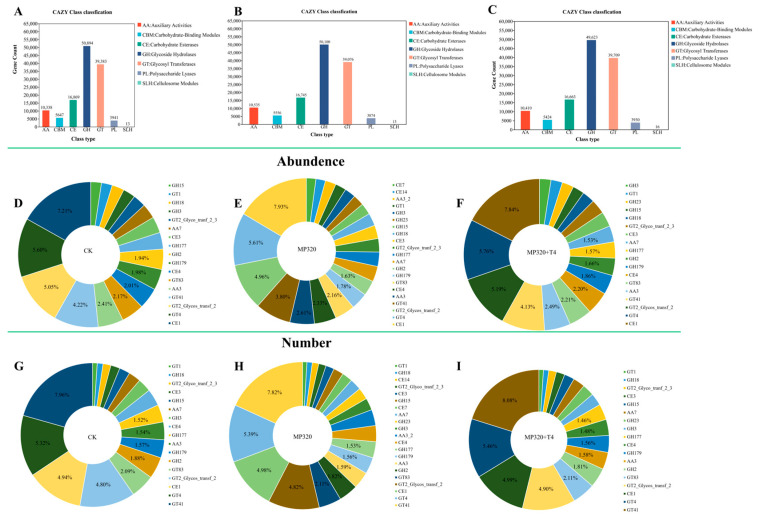
Composition of CAZY diversity in different treatment groups. (**A**) Statistics of *CAZy* classification in CK. (**B**) CAZy classification in MP320. (**C**) CAZy classification in MP320+T4. (**D**) Distribution of CAZy abundance in CK. (**E**) CAZy abundance in MP320. (**F**) CAZy abundance in MP320+T4. (**G**) Number distribution of CAZy classification in CK. (**H**) Number distribution of CAZy classification in MP320. (**I**) Number distribution of CAZy classification in MP320+T4.

**Figure 16:**
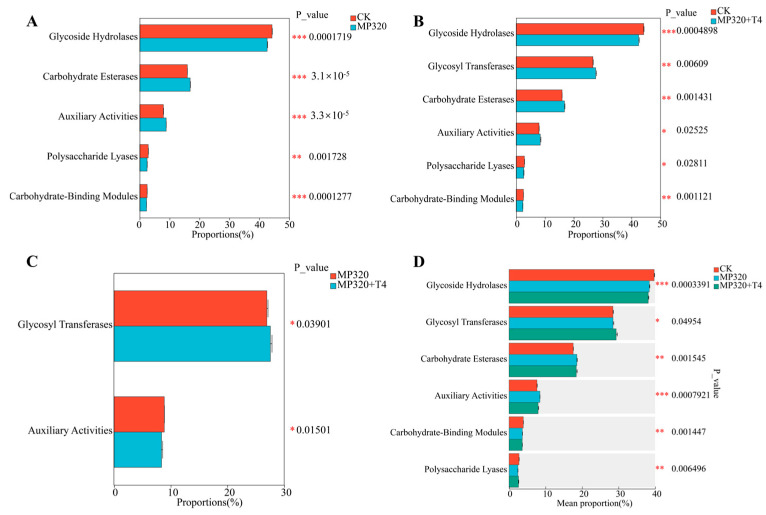
Differential analysis of CAZy abundance in different treatment groups. (**A**) Differences of CAZys composition between MP320 and CK. (**B**) Differences between MP320+T4 and CK. (**C**) Differences between MP320+T4 and MP320. (**D**) Differences in CAZys composition among CK, MP320, and MP320+T4. Values indicate the *p*-value (significant at *p*-value < 0.05) of the results of pairwise comparison using ANOVA (*: adjust *p*-value < 0.05; **: adjust *p*-value < 0.01; ***: adjust *p*-value < 0.001. This is the same below).

To characterize carbohydrate-active enzymes (CAZymes) in soil metagenomes across three treatments, 687 CAZy families were identified via comparison with the CAZy database and categorized into seven classes: auxiliary activities (AAs), carbohydrate-binding modules (CBMs), carbohydrate esterases (CEs), glycoside hydrolases (GHs), glycosyl transferases (GTs), polysaccharide lyases (PLs), and cellulosome modules (SLHs) (Supplementary Table S10; Figure 15). Glycoside hydrolases (GHs) were the most abundant class, followed by GTs, CEs, and AAs (Figure 15A–C), with similar diversity patterns across treatments (Figure 15D–F). All groups showed consistent trends in class abundance (Figure 15G–I), with the CE1 family (CE class) being the most prevalent, followed by GT4, GT2_Glycos_transf_2, and GT41. These four families accounted for 22.08% (CK), 22.3% (MP320), and 22.92% (MP320+T4) of total CAZy abundance (Figure 15D–F). Among classes, GHs harbored the most unique CAZymes (370, e.g., GH179, GH2, GH177, GH18 families), followed by GTs (114, e.g., GT4, GT2_Glycos_transf_2, GT41) and PLs (88) (Figure 15G–I). Statistical analyses revealed significant differences in five classes between MP320 and the CK, two classes between MP320+T4 and MP320, and six classes between MP320+T4 and the CK (Figure 16A–C). Crossgroup comparisons showed significant variations in all CAZy classes except SLHs (Figure 16D).

### 2.10. Diversity of ARGs, CAZy, VFs, and Genera Across Metagenomes

**Figure 17:**
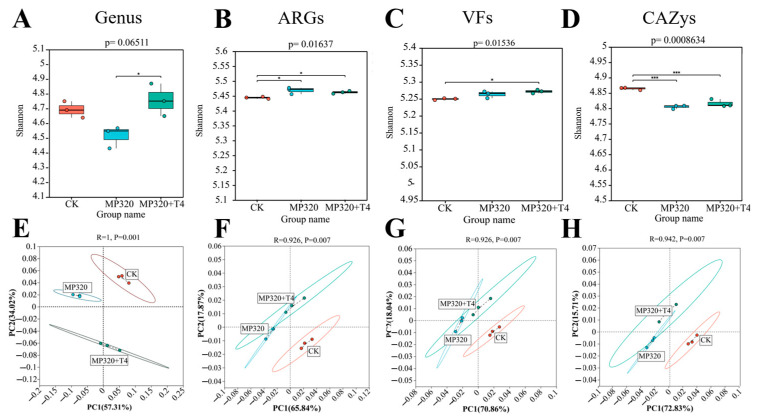
Differences in the diversity index of microbial community genera and functional genes among different treatment groups. (**A**) Comparison of the Shannon index at the genus level in different treatments. (**B**) Comparison of the Shannon index of ARGs. (**C**) Comparison of the Shannon index at the VFs. (**D**) Comparison of the Shannon index of CAZys. (**E**) PCoA based on genus level. (**F**) PCoA based on ARGs. (**G**) PCoA based on VFs. (**H**) PCoA based on CAZys. Values indicate the *p*-value (significant at *p*-value < 0.05) of the results of pairwise comparison using ANOVA (*: adjust *p*-value < 0.05; ***: adjust *p*-value < 0.001. This is the same below).

Cross-group comparisons of ARGs, CAZymes, VFs, and genus-level microbial communities (CK, MP320, MP320+T4) revealed distinct diversity patterns. *T. harzianum* T4 addition increased microbial genus diversity relative to both MP320 and the CK, with the lowest diversity observed in MP320 (Figure 17A). Consistently, ARG Shannon diversity was higher in MP320 and MP320+T4 vs. CK, with similar trends in VF diversity (Figure 17B,C). In contrast, aged microplastics reduced CAZy alpha diversity, which was partially restored by T4 co-treatment (Figure 17D). These results highlight the positive regulatory effect of *T. harzianum* T4 on the diversity of soil microbial functions and communities.

Principal coordinate analysis (PCoA) of microorganisms, ARGs, VFs, and CAZys revealed distinct clustering patterns across the three groups. At the genus level, both aged microplastics and *T. harzianum* T4 significantly altered microbial community composition (Figure 17E). Notably, ARGs, VFs, and CAZys showed significant spatial separation along PC1 and PC2 axes among the CK, MP320, and MP320+T4 (Figure 17F,G). Adonis analysis confirmed strong correlations between PC1 and PC2 (R > 0.9, *p* < 0.01), with tight intragroup clustering reflecting high sample similarity. MP320 and MP320+T4 exhibited partial overlap and distinct separation, indicating that *T. harzianum* T4 introduces unique functional shifts while retaining some similarities to the MP320 group.

### 2.11. Shared ARGs, VFs, CAZys, and Genera Among Different Treatments

**Figure 18:**
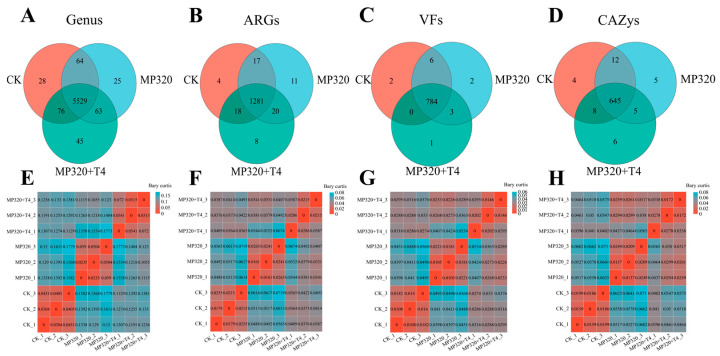
Comparative analysis of the diversity of microbial genera and functions among different treatment groups and the heatmap of similarity matrices. (**A**) Venn of microbial genera. (**B**) Venn of ARGs. (**C**) Venn of VFs. (**D**) Venn of CAZys. (**E**) Similarity matrix heatmap of genera. (**F**) Heatmap of ARGs. (**G**) Heatmap of VFs. (**H**) Heatmap of CAZys based on Bray–Curtis’s distance.

A comprehensive analysis of ARGs, VFs, CAZys, and microbial genera across the CK, MP320, and MP320+T4 samples was performed using the Bray–Curtis index. Results showed significant overlaps in taxonomic and functional compositions: 5529 genera, 1281 ARGs, 784 VFs, and 645 CAZys were shared among all groups (Figure 18A–D), comprising the core microbial functional pool. *T. harzianum* T4 co-treatment with aged microplastics introduced only 8 ARGs, 1 VF, and 6 CAZys uniquely to MP320+T4, while adding 45 genera, suggesting potential linkages between specific taxa and functional traits (Figure 18A–D). Bray–Curtis distances revealed distinct intergroup variations: genus (0.13), ARG (0.04), VF (0.02), and CAZy (0.03) profiles showed significant divergence, with MP320 and MP320+T4 exhibiting closer similarity than to the CK (Figure 18E–H).

### 2.12. Contribution and Correlation Analysis of Species to ARGs, VFs, and CAZys

The top 10 most abundant ARGs primarily included antibiotic-resistant *fabG*, ATP-binding cassette (ABC) antibiotic efflux pumps, daptomycin-resistant *liaR*, and serine/threonine kinase families. Except for Functions 01 and 07, all other resistance mechanisms involved antibiotic efflux, highlighting the dominance of ABC transporter-related genes. Function 01 (antibiotic-resistant *fabG* family) and Functions 02–04 (antibiotic efflux pump families), *Streptomyces* and *Actinomadura*, contributed substantially. For example, in Function 03, *Streptomyces* accounted for 31.25% (CK), 31.69% (MP320), and 30.69% (MP320+T4), while *Actinomadura* contributed 4.95%, 5.79%, and 5.08% across the same groups, with observable intergroup fluctuations validated by heatmap analysis (Supplementary Figure S2). Functions 05 (MFS antibiotic efflux pump family) and 06 (daptomycin-resistant liaR family) showed shifted contribution patterns. *Actinoallomurus* increased from 7.82% (CK) to 8.94% (MP320), while adding *T. harzianum* T4 slightly decreased the proportion (8.01%) compared to MP320. Meanwhile, *Streptomyces* decreased from 30.59% (CK) to 29.56% (MP320+T4). In Functions 07 (serine/threonine kinase family)-10 (antibiotic efflux pump family), complex species contributions were observed. For instance, *Streptantibioticus* contributed 3.01–5.2% in Function 07, while Function 10 involved *Streptomyces*, *Actinomadura*, *Actinoallomurus*, *Streptantibioticus*, and *Pseudonocardia* as major contributors, with *Devosia*, *Nocardia*, and others playing secondary roles. *Streptomyces* dominated across multiple functions, emerging as a key contributor to microbial community resistance mechanisms, while species like *Nocardia* and *Devosia* played minor roles. Heatmap analysis of Functions 05–06 further confirmed *Streptomyces*’s pivotal role in these resistance pathways.

In terms of VFs, the top 10 abundance functions involve microbial movement, biofilm formation, nutrient uptake, regulation of cell wall viscosity, and promotion of cell void formation to facilitate movement, adhesion, and invasion. Across the CK, MP320, and MP320+T4 groups, significant variations in species contribution proportions were observed for each function. For instance, in Function 01 (Polar flagella), *Streptomyces* accounted for 23.38% (CK), 24.33% (MP320), and 23.31% (MP320+T4), demonstrating how different treatments altered the microbial community structure and, consequently, species contributions to VF functions (Supplementary Figure S2). Species contributions varied by function. *Streptomyces* and *Actinomadura* were key contributors to Function 03 (Trehalose-recycling ABC transporter), whereas *Actinoallomurus* played a more prominent role in Function 05 (Beta-haemolysin/cytolysin), highlighting that distinct VF functions rely on specific species combinations. *Streptomyces* emerged as the dominant contributor across most functions. In Function 08 (MymA operon), *Streptomyces* represented 23.31% (CK), 23.13% (MP320), and 22.73% (MP320+T4). In contrast, *Nocardia* and *Devosia* consistently contributed less than 5% across functions, indicating minimal involvement in VF expression (Supplementary Figure S2). Collectively, bar charts and heat maps confirmed that treatments modified microbial community composition and functional contribution patterns, with *Streptomyces*, *Actinomadura*, and *Actinoallomurus* as major contributors and *Nocardia* and *Devosia* playing minor roles.

Analysis of the top 10 carbohydrate-active enzymes (CAZys) revealed functions dominated by three enzyme classes: carbohydrate esterases, glycoside hydrolases, and glycosyl transferases, with *Streptomyces* and *Actinomadura* as major contributing species. Intergroup comparisons showed distinct species contribution ratios across functions. For example, *Streptomyces* contributed 15.43% (CK), 18.31% (MP320), and 15.8% (MP320+T4) in Function 01, illustrating how microplastic and *T. harzianum* T4 treatments altered microbial community structure and functional contributions (Supplementary Figure S2). Species contribution patterns varied by function. While *Streptomyces* and *Actinomadura* were consistently significant, their proportions in Function 03 were notably lower than in other functions, highlighting that different CAZy functions rely on distinct species combinations. *Streptomyces* emerged as the predominant contributor across most functions, underscoring its central role in carbohydrate metabolic pathways.

Collective analysis of ARGs, VFs, and CAZys revealed that *Streptomyces* dominated functional contributions across most categories, with consistently high abundance ratios and contribution values. While *Actinomadura* and *Actinoallomurus* contributed to specific functions, their proportions were consistently lower than *Streptomyces*. In contrast, species like *Nocardia* and *Devosia* showed minimal functional involvement across all categories. These findings highlight *Streptomyces* as the keystone species in the microbial community, with distinct species contribution profiles across functions. The observed functional shifts underscore how treatment factors alter community structure, driving specific species combinations to mediate metabolic and resistance pathways.

### 2.13. KEGG Enrichment Analysis of Metagenomic Functional Genes

Functional gene annotation against the KEGG database identified 15,657 metabolic pathways across three treatment groups, including 297 KOs involved in carbon metabolism and 58 KOs in nitrogen metabolism. Kruskal–Walli’s rank sum tests (*p* < 0.05) with Tukey–Kramer correction (Q = 0.95) identified 2365 significantly differentially abundant KOs, spanning pathways such as microbial metabolism in diverse environments, amino acid biosynthesis, quorum sensing, pyruvate metabolism, amino sugar/nucleotide sugar metabolism, fatty acid metabolism, and glyoxylate/dicarboxylate metabolism. Key genes included *rpoE* (RNA polymerase sigma-70 factor), *qor* (NADPH: quinone reductase), *acd* (acyl-CoA dehydrogenase), and *fadD* (long-chain acyl-CoA synthetase) (Supplementary Figure S3). For nitrogen metabolism, 14 significant KOs were identified, primarily involving alanine/aspartate/glutamate metabolism, glyoxylate/dicarboxylate metabolism, and purine metabolism, with representative genes *cynT* (carbonic anhydrase), *gdhA* (glutamate dehydrogenase), and *nrtB* (nitrate/nitrite transporter). In carbon metabolism, 14 KOs were significant, covering carbon fixation, glyoxylate/dicarboxylate metabolism, pyruvate/methane/butanoate metabolism, and lipoic acid metabolism, featuring *atoB* (acetyl-CoA C-acetyltransferase), *fdoG* (formate dehydrogenase), and *frmA* (hydroxymethylglutathione dehydrogenase) (Supplementary Figures S3 and S4). Genus-level contribution analysis revealed that *Streptomyces*, *Actinomadura*, *Actinoallomurus*, and *Pseudonocardia* contributed to diverse metabolic pathways (e.g., microbial metabolism, two-component systems, ABC transporters), while *Phenylobacterium* played a key role in nitrogen metabolism-related amino acid biosynthesis. *Caulobacter* specifically contributed to carbon fixation and pyruvate metabolism (Supplementary Figure S4).

### 2.14. Correlation Analysis Between Multiple Indicators and Agronomic Traits

**Figure 19:**
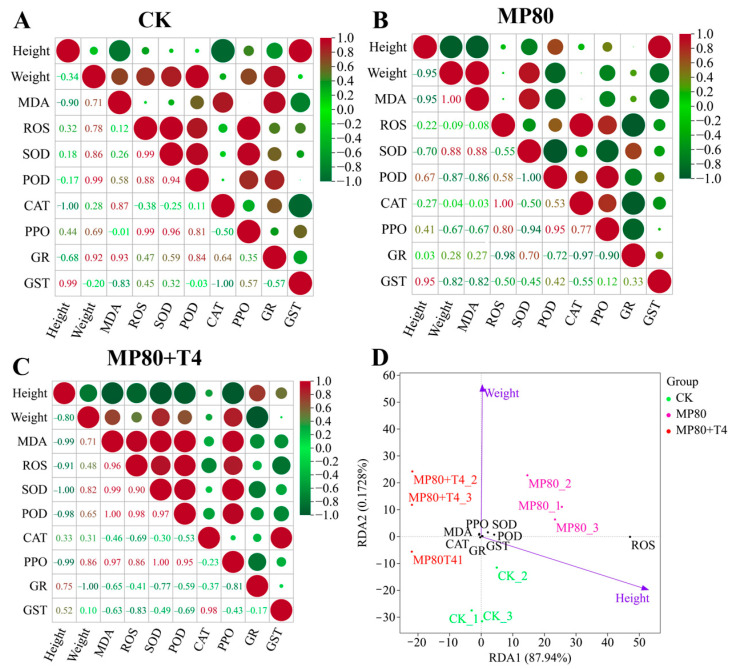
Correlation analysis and RDA between agronomic traits and enzyme activities in low-concentration aged microplastics and *T. harzianum* T4 treatment. (**A**) Correlation analysis between agronomic traits and enzyme activities in CK. (**B**) Correlation analysis of MP80. (**C**) Correlation analysis of MP80+T4. (**D**) RDA between agronomic traits, enzyme activities, and samples.

Correlation analysis and RDA analysis were conducted on agronomic traits, stress-related enzyme activities, differentially expressed genes (DEGs) identified by transcriptome screening, dominant bacterial genera selected by metagenomic analysis, AGR, virulence factors (VFs), carbohydrate-active enzymes (CAZys), and other indicators across different treatment groups. Correlation analysis revealed a basic correlation pattern among various indicators (height, weight, MDA, ROS, antioxidant enzymes, etc.) of the control group (CK). In the control group (CK), height was strongly positively correlated with the detoxification enzyme GST (r = 0.99) and strongly negatively correlated with the antioxidant enzyme CAT (r = −1.00), reflecting the precise regulation of plant height growth and the detoxification antioxidant enzyme system under blank conditions (Figure 19A). In addition, height was strongly negatively correlated with the membrane damage index MDA (r = −0.90), reflecting the negative feedback of basal growth stress injury. There is a strong positive correlation between weight and antioxidant enzyme POD (r = 0.99), indicating that the antioxidant system dominated by POD plays a core supporting role in maintaining weight biomass. At the same time, weight is significantly positively correlated with MDA (r = 0.71), suggesting that under basic conditions, weight accumulation is accompanied by certain natural membrane damage, constructing a synergistic steady state (Figure 19A). In the low concentration microplastic group (MP80), there was a strong negative correlation between height and weight (r = −0.95), as well as MDA (r = −0.95), reflecting the deep binding between plant height growth and biomass accumulation, and membrane damage formation under aged microplastic stress (Figure 19B). Weight is negatively correlated with antioxidant enzymes SOD (r = −0.09) and POD (r = −0.87), indicating that microplastic stress breaks the positive support of antioxidant enzymes for weight and the antioxidant system regulates passively (Figure 19B). Adding *Trichoderma* to the aged microplastics treatment (MP80+T4), height was strongly negatively correlated with SOD (r = −1.00) and POD (r = −0.98). *Trichoderma* induced a shift in antioxidant strategy, prioritizing stress resistance (Figure 19C). Weight is positively correlated with SOD (r = 0.82) and POD (r = 0.65) and positively correlated with MDA (r = 0.71). *Trichoderma* repairs the positive correlation between antioxidant enzymes and weight and reconstructs the relationship between stress resistance and growth (Figure 19C). In the high concentration aged microplastic group (MP320), the negative correlation between height and the WRKY70 gene was enhanced, and the positive correlation with dominant bacterial genera disappeared (Supplementary Figures S5 and S6). Weight was negatively correlated with the *WRKY40*/*WRKY40A* gene (originally positively correlated), and the correlation with the ARG/VF/CAZy functional pathway was disrupted, revealing the destructive effect of high concentration of microplastics on the growth regulatory network and the collapse of the correlation system. When *Trichoderma* was added to the high concentration of microplastics group (MP320+T4), the correlation between height and the *WRKY70* gene and the *CYPR4* gene returned to the CK group (Supplementary Figure S7). The positive correlation between weight and the WRKY40/WRKY40A gene was restored, and a strong positive correlation (r increase) with GR enzyme activity was observed (Supplementary Figure S7). The negative correlation with dominant bacterial genera was reduced to a weak positive correlation. *Trichoderma* repaired the association network between height, weight, genes, enzyme activity, and microorganisms, transforming the association collapse under extreme stress into an orderly synergy, verifying its ecological remediation potential.

**Figure 20:**
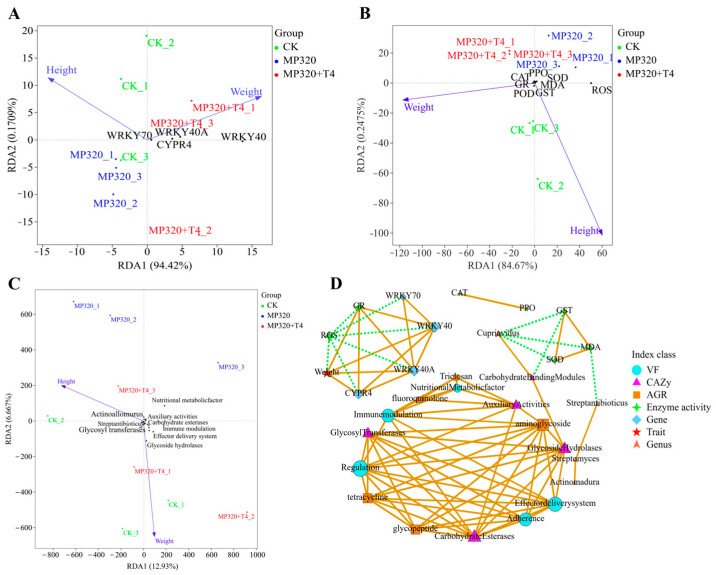
RDA and network between agronomic traits and enzyme activities of high-concentration aged microplastics treatment and *T. harzianum* T4. (**A**) RDA between agronomic traits and genes. (**B**) RDA between agronomic traits and enzyme activities. (**C**) RDA between agronomic traits and microbial function. (**D**) Networks of different indicators in the three group samples.

Redundancy analysis (RDA) showed significant distribution differences in multidimensional indicators such as genes, enzyme activity, microorganisms, and functional pathways among different treatment groups. Redundancy analysis (RDA) of low-concentration aged microplastics (MP80) related treatments showed that RDA1 accounted for 87.94% of the variance (Figure 19D). In terms of sample distribution, the CK group distribution reflects the synergistic growth and stress resistance indicators of the blank group. The MP80 group is distributed on the right side, reflecting the synergistic shift of growth oxidative damage induced by aged microplastic stress. The MP80+T4 group is located between the CK and MP80, close to the enzyme activity and membrane damage indicators, indicating that *T. harzianum* T4 regulates the antioxidant membrane damage pathway, reshapes growth and stress resistance indicators, alleviates aged microplastic stress, and height is positively correlated with the MP80 group samples, and weight is positively correlated with the MP80+T4 group samples. Enzyme activity and membrane damage are closely related to the CK and MP80+T4 group samples, verifying the alleviating effect of *Trichoderma* (Figure 19D). In the high-concentration aged microplastics treatment, the RDA1 of genes and growth indicators is 94.42% (Figure 20A). The MP320 group is separated from the CK group, while the MP320+T4 group deviates from the MP320 group and approaches the CK group. Height is biased towards the CK, and weight is strongly correlated with the MP320+T4. The distribution of samples driven by genes such as WRKY70/40A indicates that *T. harzianum* T4 can improve biomass by regulating the gene network (Figure 20A). The RDA1 of enzyme activity and stress resistance indicators is 84.67%. Samples were separated from oxidative stress indicators (MDA, ROS) and antioxidant system-related enzymes (SOD, POD, etc.), and the weight response was between the CK and MP320+T4, revealing that *T. harzianum* T4 has the function of synergistically regulating the growth and stress resistance physiological network of *N. benthamiana* (Figure 20B). The RDA1 of microbiota and functional pathways and growth indicators is 12.93%, indicating that the microbial community (such as *Actinoallomurus*) and functional genes (ARGs, CAZys) in the MP320 group are distributed discretely (Figure 20C). The MP320+T4 group approaches the CK by enriching beneficial microbial communities and activating carbon metabolism pathways (such as CAZys), alleviating the stress effect. Through network correlation analysis of agronomic traits, genes, enzyme activity, dominant bacteria, and functional pathways, under the treatment of *T. harzianum* T4, WRKY transcription factor (*WRKY40*/*70*) directly clears oxidative damage (ROS) by activating antioxidant enzymes (GR, GST) and optimizes nutrient utilization through enrichment of functional microorganisms such as Cupriavidus and Streptomyces, in conjunction with the carbohydrate metabolism pathway (CAZy), and feedback promotes plant growth (Figure 20D). Ultimately, a “gene enzyme microorganism” collaborative network will be constructed to reshape the plant microorganism interaction balance under microplastic stress.

## 3. Discussion

### 3.1. Regulation Mechanism of T. harzianum on Oxidative Stress Under Microplastic Stress

PBAT biodegradable plastic film, widely used in agriculture, can still pose environmental risks through aged microplastics, which induce plant oxidative stress (e.g., reactive oxygen species (ROS) accumulation, membrane lipid peroxidation) and disrupt soil ecology [39,40]. This study showed that aged PBAT microplastics significantly induced ROS accumulation in *N. benthamiana*, with superoxide anion levels increasing by 1.88–2.13-fold (*p* < 0.001) and malondialdehyde (MDA) content rising by 5.43–7.36-fold (*p* < 0.01) compared to the control (CK), confirming that PBAT microplastics damage plants by disrupting redox balance. These findings align with previous studies. BIO microplastics trigger oxidative damage to plant membranes and proteins while inducing antioxidant enzymes (e.g., GST, CAT, SOD) as adaptive responses [41]. Microplastics can also inhibit enzymes like acetylcholinesterase and CAT, interfering with plant metabolism [42].

Microorganisms are crucial for plastic pollution remediation, with mechanisms including attachment, mycelial growth, and enzymatic degradation of polyethylene by *Aspergillus* and *Penicillium*, ultimately decomposing it into CO_2_ and water [43,44,45]. This study further confirms that *T. harzianum* T4 accelerates degradation of PBAT films and microplastics, outperforming high-pressure sterilization and polyethylene surface sterilization in efficiency [36,37]. When combined with *Aspergillus niger*, *T. harzianum* induces significant physical changes in polyethylene terephthalate (PET) [38], highlighting its potential in composite microbial systems. We confirmed that *T. harzianum* T4 mitigates microplastic-induced oxidative damage by enhancing plant antioxidant defenses. In the MP320+T4 group, superoxide anion and MDA levels decreased to 1.47% and 29.15% of the MP320 group, respectively, accompanied by upregulation of *SOD*, *POD*, and *CAT* genes. GR activity increased by 58.88% compared to MP320, indicating enhanced ROS scavenging [41,46]. Bacterial inoculation has been shown to alleviate plant oxidative stress, particularly under low microplastic concentrations [47]. This study first demonstrates the repair capacity of *T. harzianum* under high microplastic stress, expanding applications of microbe–plant interactions in pollution remediation.

The stress-alleviating effect of *T. harzianum* T4 likely involves multiple pathways: direct ROS scavenging via secreted enzymes (e.g., GR, GST) and activation of antioxidant genes through regulation of WRKY transcription factors (e.g., *WRKY40*/*70*). Metagenomic analysis further shows that *T. harzianum* T4 reshapes soil microbial communities, enriching functional taxa like *Streptomyces* and *Cupriavidus* to optimize nutrient utilization via carbohydrate-active enzyme (CAZy) pathways (Figure 20D). This gene–enzyme–microbe collaborative network provides a novel theoretical basis for ecological remediation of microplastic pollution.

### 3.2. Activation of Photosynthetic Metabolism and Recovery of Growth Phenotype

Photosynthetic metabolism serves as the foundation for energy flow, material cycling, and oxygen supply in ecosystems, directly influencing plant growth and environmental regulation. Previous studies have shown that microplastics (MPs) disrupt the photosynthetic apparatus, leading to notable decreases in chlorophyll content and increases in carotenoid levels in rice seedlings [41]. Poly(lactic acid) (PLA) MPs exhibited the strongest inhibitory effect on biomass, while high-concentration MPs significantly reduced chlorophyll content [46]. In *Chlorella vulgaris*, 50–1000 mg/L MPs inhibited growth and chlorophyll *a* by 15.71–28.86% and 9.2–21.3%, respectively [48]. Exposure to MPs caused 42%, 45%, and 55% decreases in chlorophyll *a*, *b*, and total chlorophyll content, likely due to disrupted nutrient uptake and photosynthetic damage [49]. Consistently, aged PBAT microplastics (MP320) in this study reduced chlorophyll *a* content in *N. benthamiana* by 13.9% compared to the control (CK), confirming MP-induced photosynthetic pigment degradation.

*T. harzianum* T4 reversed MP-induced inhibition by regulating photosynthetic gene expression and increasing the content of chlorophyll *a*. The chlorophyll *a* level in the MP80+T4 increased by 16.51% compared to MP80, and MP320+T4 increased by 26.08% (*p* < 0.05) versus MP320, accompanied by significant upregulation of photosynthetic antenna protein genes such as Niben261Chr13g0674004 (*Lhca*) and Niben261Chr17g0759010 (*Lhcb*), and carbon fixation genes such as Niben261Chr07g1128061 (*rbcS*) in the MP320+T4 group (Supplementary Tables S3–S5). This correlates with biomass recovery: MP320 reduced biomass by 21.17% versus CK, while MP320+T4 increased biomass by 49.34% versus MP320, indicating enhanced photosynthetic efficiency provides energy to counteract MP stress. The phenotype of inducing an increase in chlorophyll *a* content and biomass by adding *T. harzianum* T4 was consistent in moderate concentration aging microplastic treatment (MP80+T4). Under moderate MP80 stress (80 mg/kg), the efficient degradation of microplastics by *T. harzianum* T4 renders its growth-promoting effects more prominent. The photosynthetic promotion of *Trichoderma* has been documented in multiple crops. *T. harzianum* strain T-22 enhanced tomato growth by increasing plant height, total chlorophyll content, and gas exchange parameters (e.g., net photosynthetic rate, stomatal conductance) [50]. Endophytic *Trichoderma* strains enhance photosystem activity [51]. This study first demonstrates that *Trichoderma* relieves MP stress in *N. benthamiana* by remodeling photosynthetic gene expression networks, expanding its application in stress-induced photosynthetic repair. Transcriptome data show that MP320+T4 not only upregulates photosynthetic genes but also activates glycolysis, such as Niben261Chr02g0024003 (*HXK3*), Niben261Chr03g1103005 (*HK*), and Niben261Chr13g1056008 (*PFK*), and tricarboxylic acid cycle, such as Niben261Chr03g0853016 (*PDH*) genes, indicating *Trichoderma* optimizes energy allocation via a “photosynthesis–carbon metabolism” coupling pathway (Supplementary Tables S3–S5). This mechanism aligns with *Trichoderma*-induced photosynthetic–respiratory coupling in *Cucurbita pepo* seedlings [52], providing new evidence for microbial–plant energy regulation networks.

### 3.3. The Regulatory Role of the WRKY Transcription Factor Network

WRKY proteins specifically bind to the W-box (TTGACC/T) in target gene promoters, regulating defense and environmental stress responses. The Arabidopsis WRKY family comprises 74 members, with some involved in biotic and abiotic stress responses. *TcWRKY53* is highly homologous to *AtWRKY53*, whose expression is strongly induced by stresses (e.g., NaCl, drought, cold), and signaling molecules like salicylic acid (SA) [53]. *AtWRKY6* and *AtWRKY42* mediate Arabidopsis low-phosphorus stress responses by regulating *PHO1* expression [54], while overexpression of maize *ZmWRKY33* activates stress genes (e.g., *RD29A*) and enhances salt tolerance [55], indicating conserved roles of WRKYs in cross-species stress responses. WRKYs regulate plant immunity by integrating hormone signals. Some members bind promoters of key genes in salicylic acid (SA) and jasmonic acid (JA) biosynthesis pathways to mediate pathogen defense [56,57]. For instance, *NbWRKY40* enhances resistance to tomato mosaic virus via the SA pathway in *Nicotiana benthamiana* [58]. Under oxidative stress, WRKYs maintain redox balance by regulating ROS synthesis and scavenging genes [59,60]. *TaWRKY10* activates *SOD* and *CAT* expression to alleviate drought-induced oxidative damage [59], while *OsWRKY28* enhances rice salt tolerance by directly binding the *OsDREB1B* promoter [60]. This study found that *T. harzianum* T4 treatment significantly upregulated three WRKY genes (*WRKY40*, *WRKY40A*, *WRKY70*) in the MP320+T4 group, with *WRKY40* expression increasing 2.3-fold versus MP320 (RT-qPCR validation). Co-expression analysis showed these genes were enriched in carbohydrate metabolism and pectin degradation pathways, suggesting mechanisms for alleviating microplastic stress. *WRKY40* may bind promoters of *SOD*, *POD*, and other antioxidant enzyme genes (consistent with the 58.88% increase in GR activity), mitigating microplastic-induced structural damage, which aligns with the enrichment of carbohydrate esterases (CEs) in the CAZy pathway (Figure 15). This finding first reveals that *T. harzianum* reshapes plant antioxidant systems and cell wall metabolism via the WRKY network, providing new targets for bioremediation of microplastic stress.

### 3.4. Reshaping of Soil Microbial Community Structure and Function

Microplastics have been detected in various environmental matrices (water, sediments, soil, plants, animals), with soil recognized as the primary reservoir [61]. Upon entering soil, microplastics alter nutrient–enzyme ratios, forming plastic spheres that distinctively impact soil microbial community structure [62]. Studies show that microplastics like PVC, LDPE, and PE reduce soil bacterial diversity, with inhibitory effects escalating with microplastic abundance [63,64]. High-density polyethylene (HDPE) microplastics further affect microbial diversity by decreasing soil pH [65]. Therefore, MPs have an impact on microbial communities and diversity [62], nutritional metabolism [66], and enzyme activity [67]. This research confirms that microplastic stress increases the abundance of *Actinobacteria* (47.36%) and *Proteobacteria* (30.76%) while reducing *Bacteroidota*, indicating selective screening of the soil microbiome. Microbial colonization alters microplastic surface density, promoting the enrichment of degrading bacteria (e.g., *Pseudomonas* and *Burkholderia*) and accelerating biodegradation. Functional gene analysis shows significant enrichment of tetracycline resistance gene *tetA5* and multidrug resistance gene *MDR* (31.5–40.7% increase) in microplastic-treated soils, potentially linked to microplastics serving as vectors for horizontal gene transfer of resistance genes. However, *T. harzianum* T4 reverses this trend, reducing ARG abundance by 28–35% in MP320+T4, possibly via niche competition or antimicrobial secretion. Co-treatment with *T. harzianum* T4 restores microbial alpha diversity (Chao index increased by 3.1% more than MP320) and enriches Bacteroidota (6.8%) and Myxococcata (5.1%), which are associated with organic matter decomposition. *T. harzianum* significantly upregulates carbohydrate-active enzyme (CAZy) genes, with glycoside hydrolase (GH) and glycosyltransferase (GT) families increasing by 16–22% compared to MP320, enhancing soil carbon metabolism and microplastic degradation potential. The activation of CAZy pathways positively correlates with the enrichment of functional taxa like *Streptomyces* (*r* = 0.72, *p* < 0.01), validating a “microbe–enzyme system” for synergistic degradation (Figure 15).

This study first reveals that *T. harzianum* alleviates microplastic stress through a dual mechanism of plant physiological regulation–soil microbial functional remodeling by enhancing plant antioxidant systems via WRKY transcription factor regulation and reconfiguring microbial communities to activate brbon metabolism and suppress resistance genes. This provides a new paradigm for farmland microplastic remediation that *Trichoderma* not only degrades plastic polymers but also improves soil health for sustainable restoration.

### 3.5. Linkage Between T. harzianum-Mediated PBAT Enzymatic Degradation and Plant Physiological Benefits

The ability of *T. harzianum* T4 to mitigate microplastic stress and promote plant growth stems from its intrinsic capacity to enzymatically degrade PBAT, which forms a direct functional link to plant physiological benefits. Previous studies have identified key enzymes secreted by *T. harzianum* involved in plastic degradation, including esterases, lipases, laccase, and manganese peroxidase [36,37,38,46]. These enzymes specifically target the ester bonds in the poly (butylene adipate-co-terephthalate) backbone of PBAT, cleaving long-chain polymers into low-molecular-weight (LMW) intermediates (e.g., adipic acid, 1,4-butanediol, terephthalic acid) and ultimately mineralizing them into CO_2_ and water [36,37]. Consistent with these findings, metagenomic data from this study show that *T. harzianum* T4 significantly upregulates CAZy enzyme families associated with ester bond hydrolysis (e.g., carbohydrate esterases [CE]) and aromatic compound degradation (e.g., glycoside hydrolases [GH]) in MP-contaminated soils (Figure 15). This enzymatic degradation process directly benefits plant physiology through alleviation of physical and chemical stress and provision of bioavailable nutrients. PBAT microplastics accumulate in the rhizosphere to physically block root pores, inhibit nutrient, and release toxic additives [13,16,17,20]. *T. harzianum*-secreted enzymes reduce the abundance of intact microplastic particles and their toxic metabolites, thereby mitigating oxidative stress, which was evidenced by superoxide anion levels down to 1.47% of the MP320 group and MDA content reduced to 29.15% of the MP320 group. This protects plant cell membranes and maintains redox balance, laying the foundation for normal physiological processes. The LMW intermediates generated by PBAT enzymatic degradation serve as readily utilizable carbon substrates for both *T. harzianum* and plant roots [36,37]. *T. harzianum* uses these carbon sources to proliferate and secrete plant growth-promoting substances, while plants absorb excess carbon to support photosynthetic metabolism, which explains the upregulation of photosynthetic antenna protein genes (*Lhca*, *Lhcb*) and carbon fixation genes (*rbcS*) in the MP320+T4 group. This carbon supply also enhances energy metabolism, as reflected by the activation of glycolysis and tricarboxylic acid cycle genes, ultimately promoting biomass accumulation (49.34% increase vs. MP320 group). Notably, the WRKY transcription factor network (*WRKY40/40A/70*) acts as a downstream mediator of this linkage. Enzymatic degradation reduces microplastic stress signals, which upregulate WRKY genes to further activate antioxidant enzymes (SOD, POD, GR) and photosynthetic gene expression which form a closed loop between PBAT degradation and plant stress tolerance.

### 3.6. Ecological and Biotechnological Implications of T. harzianum for Sustainable Microplastic Remediation

The comprehensive findings of this study, encompassing plant oxidative stress alleviation, photosynthetic metabolism recovery, WRKY-mediated signaling, and soil microbial functional remodeling, underscore the multifaceted value of *T. harzianum* T4 in addressing agricultural microplastic pollution within a broader ecological and biotechnological framework. Ecologically, microplastic pollution in farmlands is not an isolated issue but intersects with multiple global challenges, including soil degradation, food security, and antimicrobial resistance [4,5,26]. Notably, our use of environmentally realistic PBAT microplastic concentrations (80 and 320 mg/kg) aligns with field-observed accumulation levels (1 and 80,000 particles/kg) in intensive agriculture, ensuring that the stress-alleviating mechanisms identified (e.g., enzymatic degradation, WRKY-mediated antioxidant activation) are biologically relevant to real-world crop responses [68]. Aged PBAT microplastics disrupt the plant–soil–microbe symbiosis by inducing oxidative stress in crops, reducing soil microbial diversity, and promoting ARG dissemination, all of which threaten the stability of agricultural ecosystems and the safety of food chains [24,41,42]. Our results demonstrate that *T. harzianum* T4 mitigates these interconnected risks simultaneously. It enhances plant tolerance to microplastics by regulating antioxidant and photosynthetic pathways, restores soil microbial homeostasis by enriching functional taxa involved in organic matter decomposition, and suppresses the spread of ARGs. This holistic regulatory effect makes *Trichoderma* a key component of ecological intensification strategies, which aim to balance agricultural productivity with environmental sustainability. Biotechnologically, the mechanisms identified in this study provide actionable targets for translating basic research into practical remediation tools. The WRKY transcription factor network and CAZyme-mediated carbon metabolism pathway can be exploited to develop dual-purpose crops with enhanced microplastic stress tolerance and improved rhizosphere microbial recruitment. Additionally, *T. harzianum* T4 can be formulated into bioinoculants combined with organic amendments (e.g., compost, biochar) to improve its colonization and degradation efficiency in field conditions, addressing the gap between laboratory results and real-world agricultural applications. Compared to physical (e.g., sieving) or chemical (e.g., oxidizing agents) remediation methods, Trichoderma-based bioremediation is cost-effective, environmentally benign, and compatible with conventional agricultural practices (e.g., plastic film mulching, crop rotation), making it accessible for smallholder farmers and large-scale agricultural operations alike.

Future research should focus on validating these findings under field conditions, considering variables such as soil type, climate, and crop species to optimize the application of *T. harzianum* inoculants. Furthermore, exploring the synergistic effects of *T. harzianum* with other microbial strains or engineered microorganisms could enhance microplastic degradation efficiency. Additionally, investigating the long-term ecological impacts of *Trichoderma* application, such as its persistence in soil, potential effects on non-target organisms, and contribution to soil carbon sequestration, will be critical for ensuring the sustainability and safety of this bioremediation strategy.

## 4. Materials and Methods

### 4.1. The Aging Treatment of PBAT Biodegradable Plastic Film

Ultraviolet irradiation was used for the aging treatment of newly prepared pure PBAT biodegradable plastic film in a QUV accelerated weathering tester (Q-Lab Co., Ltd., Cleveland, OH, USA) with a UVA-340 lamp at an intensity of 0.89 W/m^2^ and a temperature of 60 °C. During the aging cycle, PBAT biodegradable plastic film samples were collected at 72 h. After completing sample collection, the samples were promptly transfer into specialized storage bags for subsequent performance analysis and microplastic preparation.

### 4.2. Preparation and Characterization of Aged Microplastics

The PBAT biodegradable plastic films, aged for 72 h by UV irradiation, were used for preparing aged microplastics. The aged films were frozen with liquid nitrogen, crushed using a pulverizer, and then sieved through a 300-mesh stainless-steel sieve to obtain aged microplastic particles with consistent particle sizes. The aging microplastic particles were analyzed for particle size using Kalliope (Anton Paar, Shanghai, China) according to the instrument operation manual.

### 4.3. Morphological Observation and Molecular Identification of the Fungal Strain

The degradation-functional fungal strain used in this study was isolated from PBAT biodegradable mulching films, which had previously been used to cover planted potatoes. Morphological structures of hyphae and spores of the fungal strain were observed microscopically using an optical microscope. The primer pairs ITS1 (TCCGTAGGTGAACCTGCGG) and ITS4 (TCCTCCGCTTATTGATATGC) were employed for the sequencing analysis of the fungal strain. The target sequences were retrieved using NCBI-BLAST (https://blast.ncbi.nlm.nih.gov/Blast.cgi, accessed on 24 May 2024), while phylogenetic tree construction was implemented using MEGA7 (v7.0.26).

### 4.4. Degradation of Biodegradable Plastic Films by T. harzianum

The PBAT biodegradable plastic films were cut into 15 cm × 1 cm (length × width) strips, which underwent the following sterilization process: soaked in sterile 3% KCL solution for more than 30 min, rinsed three times with sterile water, shaken with anhydrous ethanol for 15 min, rinsed three times again with sterile water, surface moisture blotted with sterile filter paper, air-dried on a super clean bench, and irradiated with ultraviolet light for 1 h. Under sterile conditions, the *T. harzianum* strain T4 was inoculated into a basic inorganic salt liquid medium containing sterile white PBAT film strips and cultured on a shaker at 150 rpm and 28 °C for 5 days. After cultivation, the surface morphology of the film strips was observed using a scanning electron microscope (FEI Phenom Prox, Phenom-world B.V., Eindhoven, The Netherlands) operating at a voltage of 15 kV. The experiment consisted of three biological replicates (*n* = 3) and included blank controls. Basic inorganic salt culture medium: Each liter of culture medium contains K_2_HPO_4_ 0.7 g, KH_2_PO_4_ 0.7 g, MgSO_4_·7H_2_O 0.7 g, NH_4_NO_3_ 1.0 g, NaCl 0.005 g, FeSO_4_·7H_2_O 0.002 g, ZnSO_4_·7H_2_O 0.002 g, MnSO_4_·H_2_O 0.001 g (Macklin, Shanghai, China). The remaining amount is distilled water. The pH was adjusted to 7.0.

### 4.5. Interaction Experiments

*Nicotiana benthamiana* was used as the model plant, and JIFFY nutrient soil (diameter = 30 mm) served as the growth substrate. The experiment followed a clear sequential workflow to ensure consistency. Firstly, high concentrations (80 mg/kg and 320 mg/kg) of the 72-hour-aged microplastics were uniformly mixed into the JIFFY nutrient soil to prepare microplastic-contaminated substrates, while blank nutrient soil without microplastic addition was used as the control substrate. Five treatment groups were established with three biological replicates each (*n* = 3): blank control (CK, nutrient soil without microplastics or *T. harzianum* T4), aged microplastic treatments (MP80: nutrient soil + 80 mg/kg aged microplastics; MP320: nutrient soil + 320 mg/kg aged microplastics), and *T. harzianum* + aged microplastic co-treatments (MP80+T4: nutrient soil + 80 mg/kg aged microplastics + *T. harzianum* T4; MP320+T4: nutrient soil + 320 mg/kg aged microplastics + *T. harzianum* T4). Secondly, fourteen-day-old *N. benthamiana* seedlings with uniform growth status were then transplanted into the pre-prepared substrates (one seedling per pot). Concurrently, *T. harzianum* T4, cultured in PDB medium for 5 days, was centrifuged to collect fungal mycelia, and diluted with sterile water to prepare a pure fungal solution. An amount of 3 mL of the fungal solution was evenly sprinkled into the soil of *N. benthamiana* in the MP80+T4 and MP320+T4 groups, while the CK, MP80, and MP320 groups were supplemented with 3 mL of sterile water to maintain consistent soil moisture conditions after transplanting for one day. All treated plants were cultured under controlled environmental conditions (temperature: 25 ± 2 °C, photoperiod: 16 h light/8 h dark, relative humidity: 60 ± 5%). After 15 days of cultivation, plant height and fresh weight were measured, and then plant tissues (roots and leaves) as well as rhizosphere soil were collected and frozen in liquid nitrogen. All samples were stored at −80 °C for subsequent omics analysis and physiological index detection.

### 4.6. Enzyme Activity of N. benthamiana

Stress-related enzyme activity analyses were performed on *N. benthamiana* plants subjected to the interaction experiment treatments. The enzymes analyzed included polyphenol oxidase (PPO), peroxidase (POD), superoxide dismutase (SOD), catalase (CAT), glutathione reductase (GR), and glutathione S-transferase (GST). For PPO, POD, SOD, and CAT detection, the kits from Shanghai Yuanye Bio-Technology Co., Ltd. (Shanghai, China) were used, including the PPO detection kit (catechol microplate method, R30313), POD detection kit (guaiacol microplate method, R30311), total SOD activity detection kit (NBT method, R33182), and CAT detection kit (R30337). GR and GST were assayed using kits from Shanghai Macklin Biochemical Technology Co., Ltd. (Shanghai, China), including the GR activity assay kit (G930919) and GST activity assay kit (G930917). All procedures were strictly performed according to the instructions of the manufacturers.

### 4.7. Superoxide Anion, H_2_O_2_, MDA, and Chlorophyll of N. benthamiana

The contents of superoxide anion, H_2_O_2_, and malondialdehyde (MDA) in *N. benthamiana* plants subjected to the interaction experiment treatments were detected using the superoxide anion free radical detection kit (sulfonamide microplate method, R30342), hydrogen peroxide (H_2_O_2_) detection Kit (titanium sulfate microplate method, R30338) from Shanghai Yuanye Bio-Technology Co., Ltd. (Shanghai, China), and malondialdehyde (MDA) content assay kit (M926324) from Shanghai Macklin Biochemical Technology Co., Ltd. (Shanghai, China), respectively.

Chlorophyll (a and b) content was determined using the ethanol extraction colorimetric method. Briefly, after removing the main vein from *N. benthamiana* leaves, approximately 0.5 g of leaf tissue was weighed and placed into a 50 mL centrifuge tube. Subsequently, 25 mL of 95% ethanol was added, and the tube was sealed and incubated at room temperature in the dark for 24–36 h to facilitate complete pigment extraction. The extract was diluted as needed, and absorbance values were measured at 665 nm, 649 nm, and 470 nm using a UV754N UV–Visible spectrophotometer (Shanghai Yidian Analytical Instruments Co., Ltd., Shanghai, China). Pigment concentrations were calculated using the following formulas:

Chlorophyll *a* (C*_a_*, mg/L): C*_a_* = 13.95 × OD_665_ − 6.88 × OD_649_

Chlorophyll *b* (C*_b_*, mg/L): C*_b_* = 24.96 × OD_649_ − 7.32 × OD_665_

Total carotenoids (C*_x_*, mg/L): C*_x_* = (1000 × OD470 − 2.05 × C*_a_* − 114.8 × C*_b_*)/245

The final pigment content (mg/g fresh weight) was calculated as follows:

Content = (C × V)/(W × 1000).

where C is the pigment concentration (C*_a_*, C*_b_*, or C*_x_* in mg/L), V is the extraction volume (25 mL), and W is the fresh weight of the sample (g).

### 4.8. RNA-Seq

Total RNA from *N. benthamiana*, subjected to interaction experiments, was extracted using TRIzol^®^ reagent (Invitrogen, Carlsbad, CA, USA) following the manufacturer’s protocol. RNA quality and concentration were evaluated using a 5300 Bioanalyzer (Agilent, Santa Clara, CA, USA) and an ND-2000 spectrophotometer (NanoDrop Technologies, Wilmington, DE, USA), respectively. High-quality RNA samples were used to construct sequencing libraries, which were sequenced on the NovaSeq X Plus platform by Majorbio (www.majorbio.com) according to standard protocols. FPKM quantified gene expression levels and differentially expressed genes (DEGs) were identified with screening criteria of log_2_FC > 2 and *p* < 0.05. Functional annotation and enrichment analysis of DEGs were performed using KEGG and GO databases, with all data mining and bioinformatics analyses conducted on the Majorbio cloud platform (https://cloud.majorbio.com/, accessed on 28 October 2024) [69].

### 4.9. QRT-PCR Verified Gene Expression

Total RNA from *N. benthamiana* plants in interaction experiments (*n* = 3) was extracted using the Quick RNA Isolation Kit (HuaYueyang, Beijing, China). Following assessments of concentration, purity, and integrity, cDNA synthesis was performed using the Hifair^®^ III 1st strand cDNA synthesis kit (Yeasen, Shanghai, China). Nine target genes were selected for qRT-PCR analysis with gene-specific primers (Supplementary Table S11), employing a LightCycler 96 Real-Time PCR System (Roche, Basel, Switzerland) and Hieff qPCR SYBR Green Master Mix (Yeasen, Shanghai, China). Relative quantification data were analyzed using the 2^−ΔΔCT^ method, with three biological replicates and *GAPDH* serving as the reference gene.

### 4.10. Metagenomic Sequencing and Analysis

Rhizosphere soil DNA was extracted using the Mag-Bind^®^ Soil DNA Kit (Omega Bio-tek, Inc., Norcross, GA, USA). Following extraction, DNA concentration and purity were assessed, and integrity was verified by 1% agarose gel electrophoresis. DNA fragmentation was performed using a Covaris M220 (Covaris LLC, Woburn, MA USA), and fragments of approximately 350 bp were size-selected for PE library construction. The NEXTFLEX Rapid DNA-Seq Kit (Bioo Scientific, Austin, TX, USA) was used to prepare the libraries, which were then sequenced on an Illumina platform by Majorbio (www.majorbio.com). Specific protocols and analytical approaches followed the previous report [70]. All data mining and bioinformatics analyses were conducted on the Majorbio cloud platform (https://cloud.majorbio.com, accessed on 28 October 2024). Three biological replicates (*n* = 3) and the Kruskal–Wallis H test were performed for metagenomic data validation and multiple comparisons.

## 5. Conclusions

This study systematically elucidates the regulatory mechanisms of *T. harzianum* T4 in *N. benthamiana* under aged microplastic stress and the corresponding soil microbial responses. High-concentration microplastics significantly inhibit plant growth, reducing plant height and biomass, and disrupt physiological functions by inducing oxidative stress and impairing the photosynthetic system. Co-treatment with *T. harzianum* T4 activates the plant antioxidant defense system, resulting in decreased superoxide anion and MDA levels, while upregulating antioxidant enzyme activities (e.g., SOD, POD). Additionally, it significantly increases chlorophyll *a* content and photosynthesis-related gene expression, restoring photosynthetic efficiency. Transcription factor regulatory networks show significant upregulation of *WRKY40* and other genes, synergistically enhancing stress tolerance. At the soil microecological level, aged microplastic stress reduces microbial alpha diversity, abnormally increases the abundance of dominant phyla (*Actinobacteria*, *Proteobacteria*), and enriches tetracycline resistance genes (*tetA5*) and multidrug resistance genes (*MDR*). *T. harzianum* T4 reshapes the microbial community structure, restores functional microbial groups, downregulates ARG abundance, and significantly activates carbohydrate-active enzymes (CAZys), thereby enhancing soil carbon metabolic functions. In conclusion, *T. harzianum* T4 effectively alleviates microplastic-induced growth inhibition and ecological toxicity through a dual pathway of plant physiological regulation and soil microbial function optimization. This study not only provides pioneering insights into the molecular mechanisms of microbe–plant interactions under aged microplastic stress but also proposes a novel bioremediation strategy for farmland microplastic pollution. The synergistic effects of *T. harzianum* T4 in degrading plastic polymers and regulating soil health highlight its agricultural application value. Future research will focus on functional validation of its degrading enzymes and their stability in field conditions to facilitate technological translation.

## Data Availability

The data used in this study include RNA-Seq profiling of *N. benthamiana* samples and metagenomic data of soil. The original analysis dataset presented in this study is included in the manuscript and Supplementary Material. Further inquiries can be directed to the corresponding author confidentially and is available upon request.

## Supplementary Materials

The following supporting information can be downloaded at: https://www.mdpi.com/article/10.3390/ijms262311667/s1.
