# Supplementary material for: Physiological and Multi-Omics Insights into Trichoderma harzianum Alleviating Aged Microplastic Stress in Nicotiana benthamiana
Source: Int J Mol Sci. 2025 Dec 2;26(23):11667. doi: 10.3390/ijms262311667 (PMC12692410; doi:10.3390/ijms262311667)
Supplement: Supplementary file 1 [file ijms-26-11667-s001.zip › Supplementary Figures and Table S11.pdf]

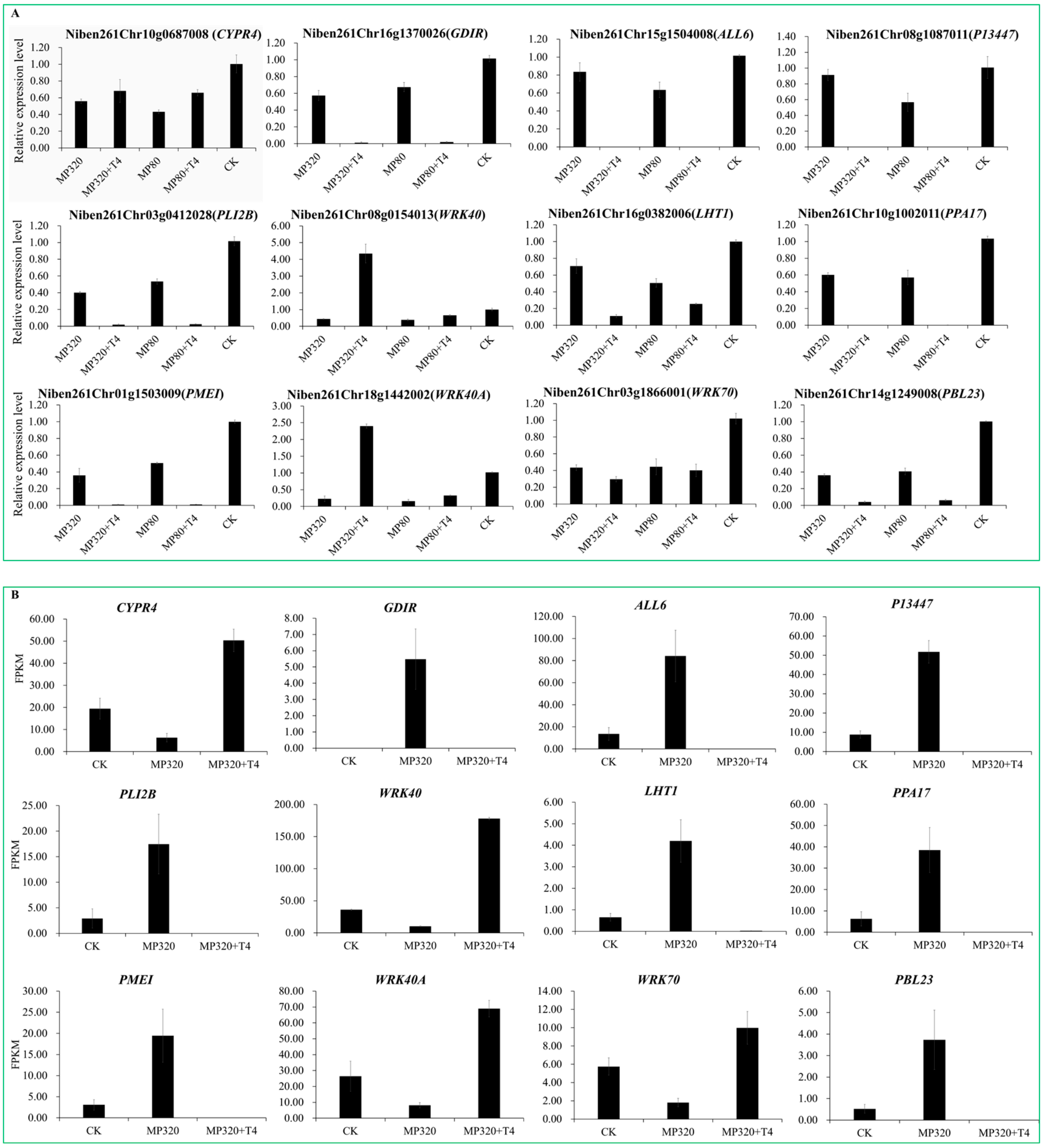

Supplementary Figure S1. qRT-PCR validation of relevant gene expression levels in different treatment groups

(A). qRT-PCR validation of relative gene expression levels; (B). FPKM of relative gene

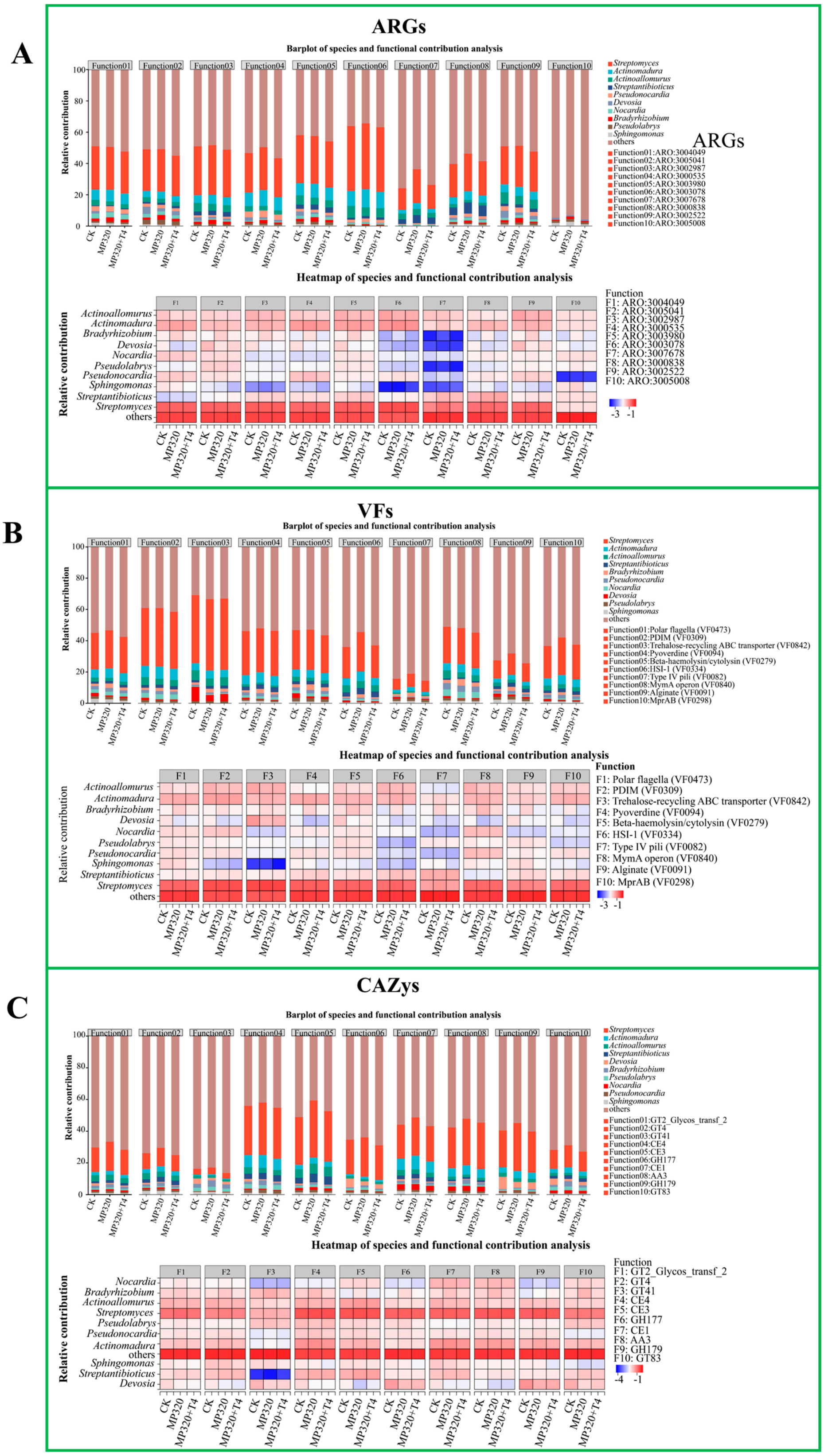

Supplementary Figure S2. Functional contributions of soil microorganisms in different aspects among different treatment groups

(A). Analysis of ARGs functional contributions; (B). Analysis of VFs' functional contributions (C). Analysis of Cazys' functional contributions

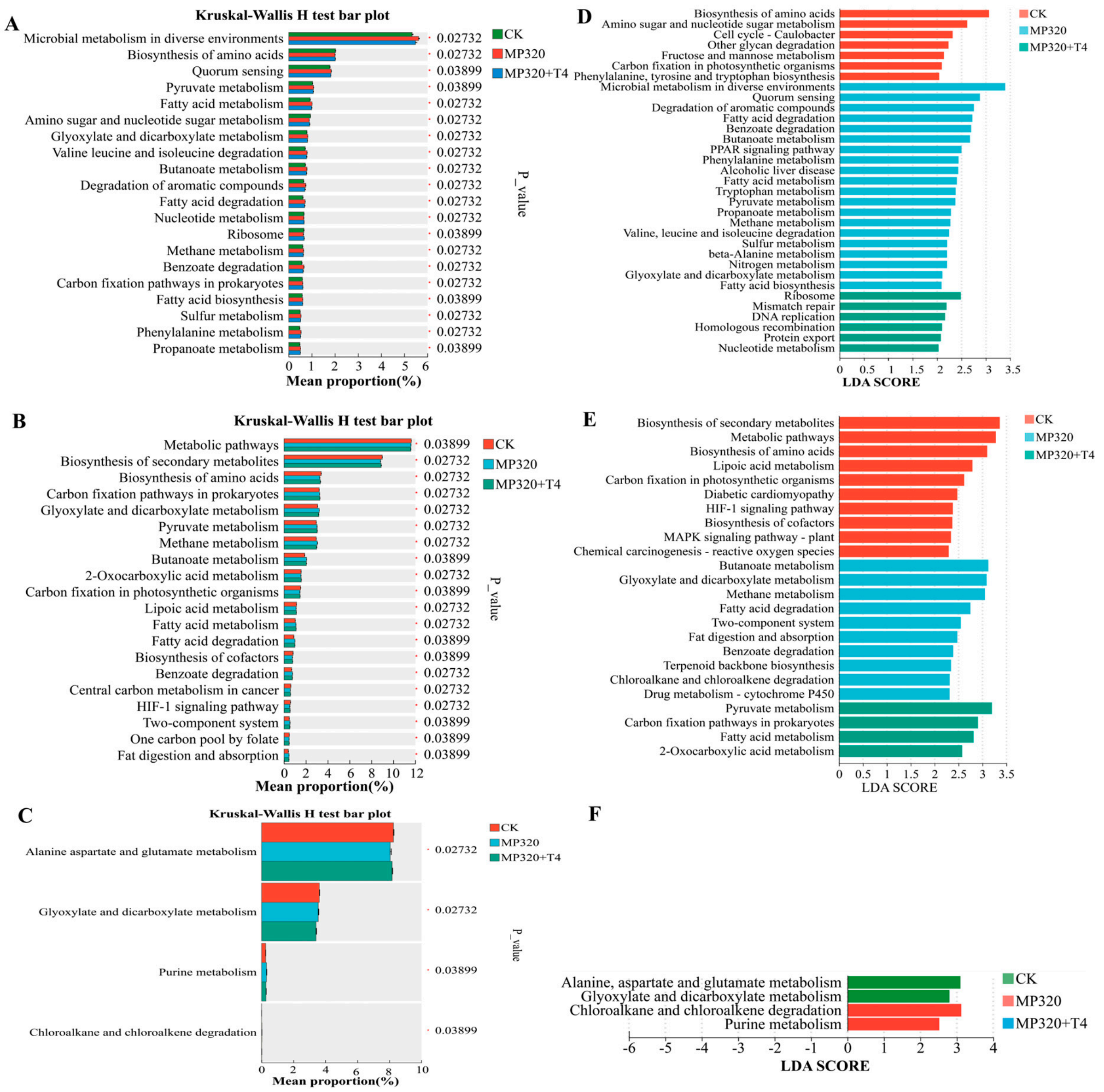

Supplementary Figure S3. Analysis of KEGG pathway differences among different treatment groups

(A). Differences in the high abundance KEGG metabolic pathways annotated between different treatments; (B). Differences in carbon metabolism between different treatments; (C). Differences in nitrogen metabolism between different treatments; (D). Differences in high abundance KEGG metabolic pathway based on LDA between different treatments; (E). Differences in carbon metabolism annotated based on LDA between different treatments; (F). Differences in nitrogen metabolism annotated based on LDA between different treatments

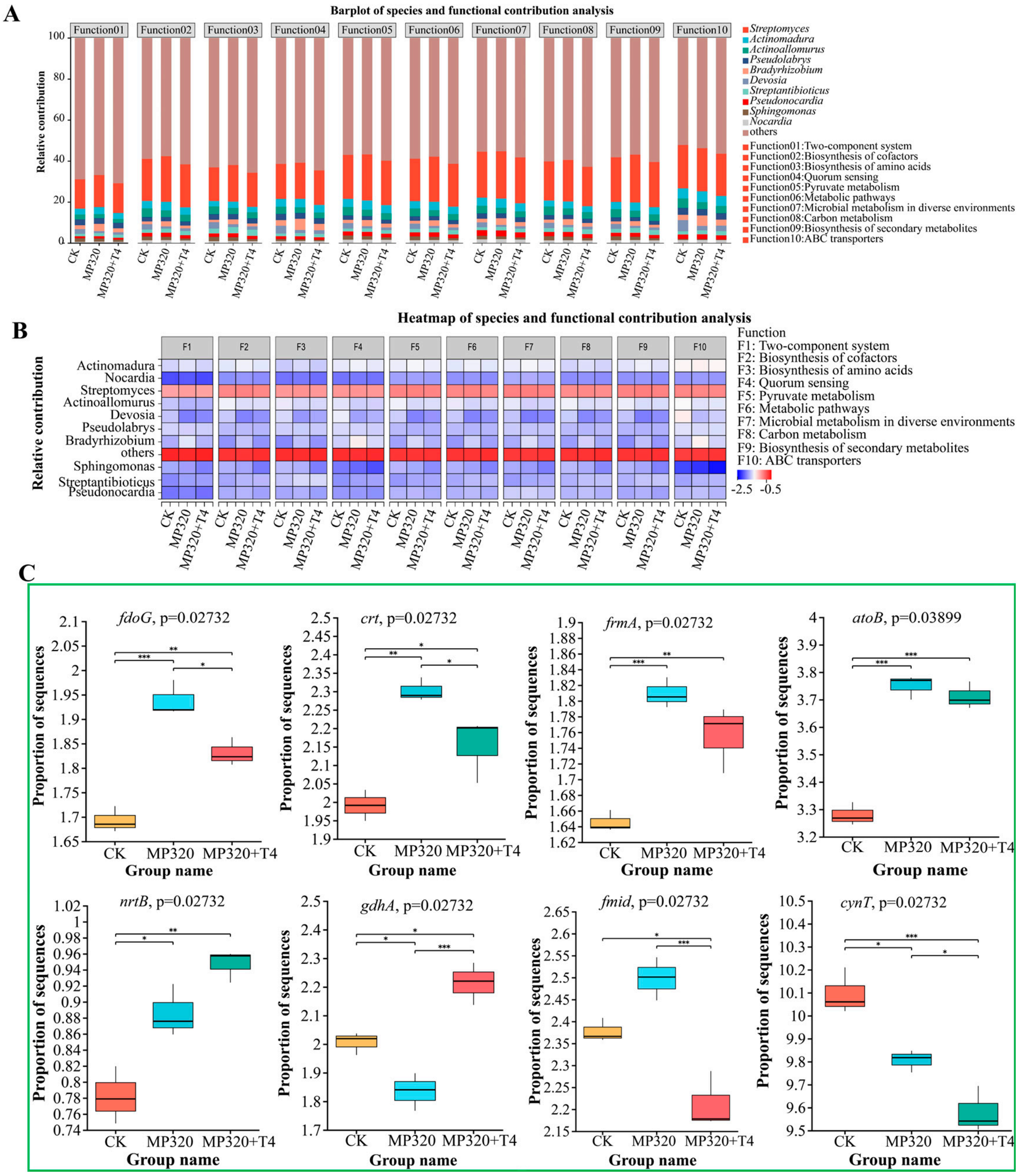

Supplementary Figure S4. Functional contributions of soil microorganisms in KEGG pathway among different treatment groups

(A). Analysis of KEGG functional contributions; (B). Heatmap of KEGG functional contributions (C). Comparison boxplot of a single species in KO among different treatment groups

Supplementary Table S11. Relevant Primers for qRT-PCR Validation

Primer	Forward (5'-3')	Reverse (3'-5')
PLI2B	CACAGGAACTTTGGATAAATGC	AAGTAACACCATCCGCAGACAAC
GDIR	GAGTGAAGAGGATGAGAAGCAAG	AGGACTGGAAATAAATGGCTCTG
PMEI	ACCAAACACAGACATTTATGCTA	ATCTTCCTCAGTCCTCTTCATTG
ALL6	GCTGAAGGTCTTGGATATACTCAT	CAAGGCTTCAAATTCTTCAAATTC
PPA17	CTGCCTTGAACATATTAGCGACA	CACATCTCCTCTCCATGCCTTTG
P13447	CCGTGTTCAATTTCAGACCAAAC	CCCTTCAACTGAGTATGTCTCCT
CYPR4	GGACAGTGCTCACAGTTGCTATC	CGACTCTCAACAACGGACTCATC
PBL23	TGATTTGTGATGTTGTTACTGCT	CGGCTCTGGTTCTGTTGTGTTAG
LHT1	TGGATTGGGAGAAGTGGCATTTG	CTCAGGTGTTGAAGGAATTGTAGC
WRK40	AGACCTCTTCGAGTTCCTGATGG	CTTGACTGGCGTGTGATCTATTC
WRK70	CGGTGATCTGAAGTCCAATGATTC	GTAACACCTTCTCTTGTAGCAAC
PRK2	CCAAATGCTCAAGTGAACCAAAG	TGGCGAAGGATGACGATAACAAC
WRK40A	GGATGAGGCAAGTGATTTATTAG	TGGTTTCTCAACGCATTGTAATTC

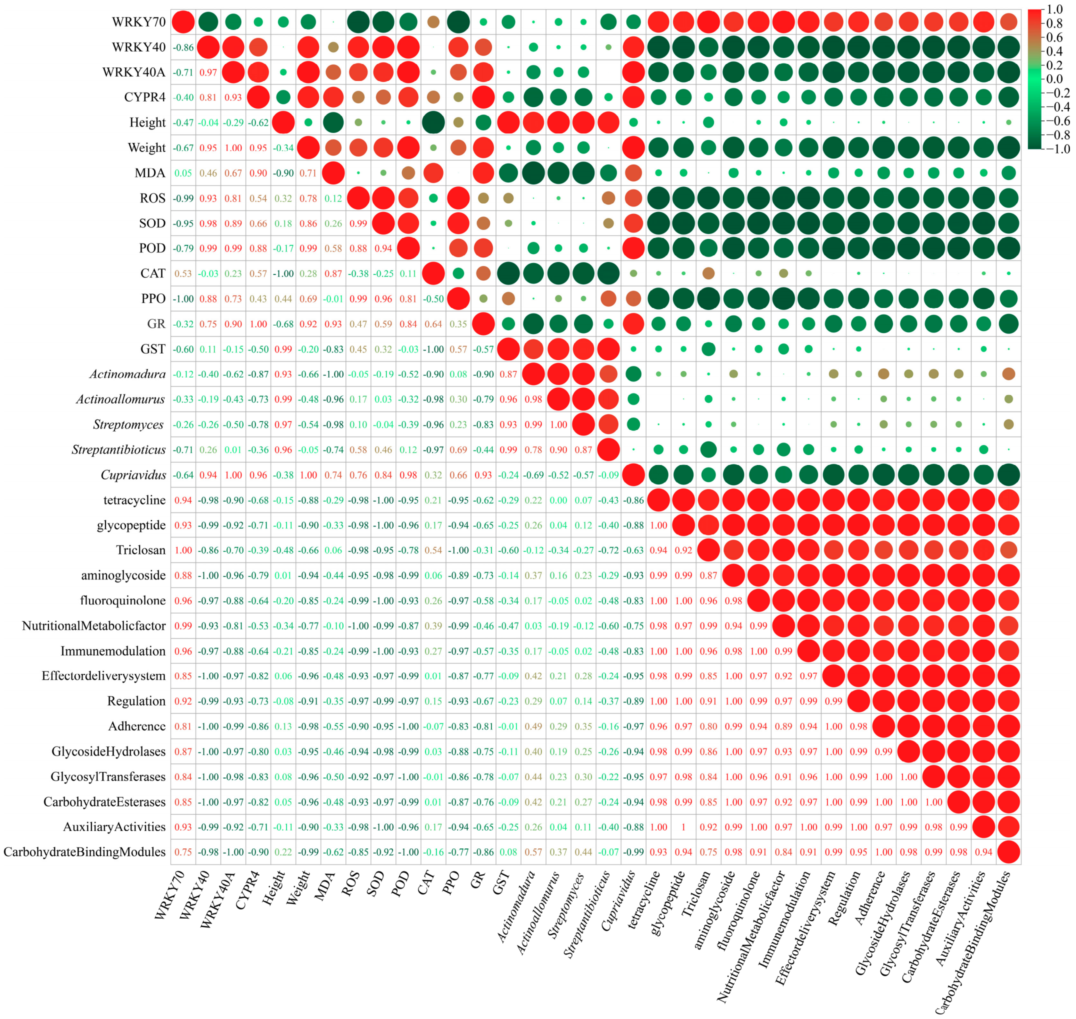

Supplementary Figure S5. Correlation analysis of *Nicotiana benthamiana* related indicators in the CK group

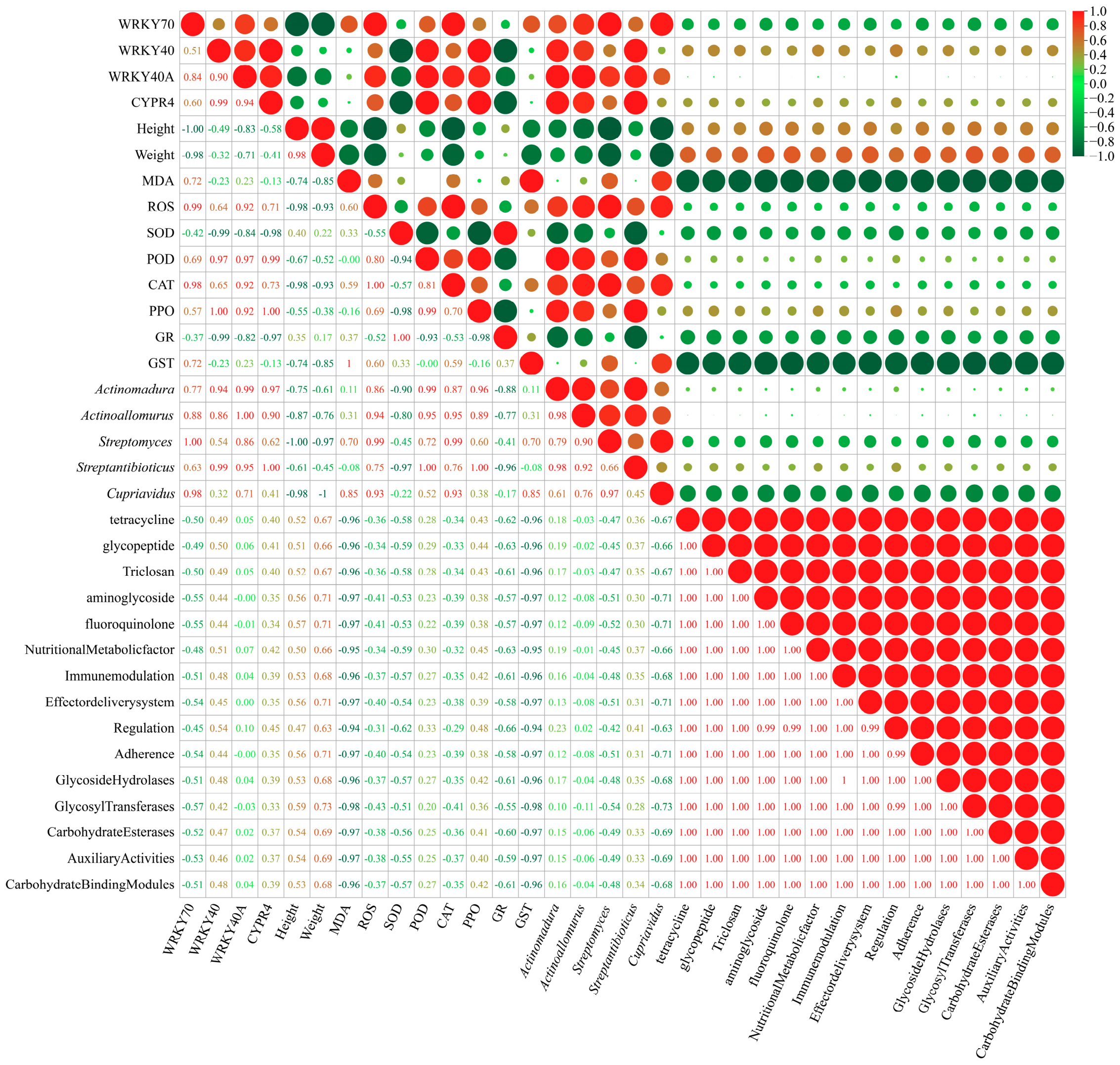

Supplementary Figure S6. Correlation analysis of *Nicotiana benthamiana* related indicators in the MP320 group

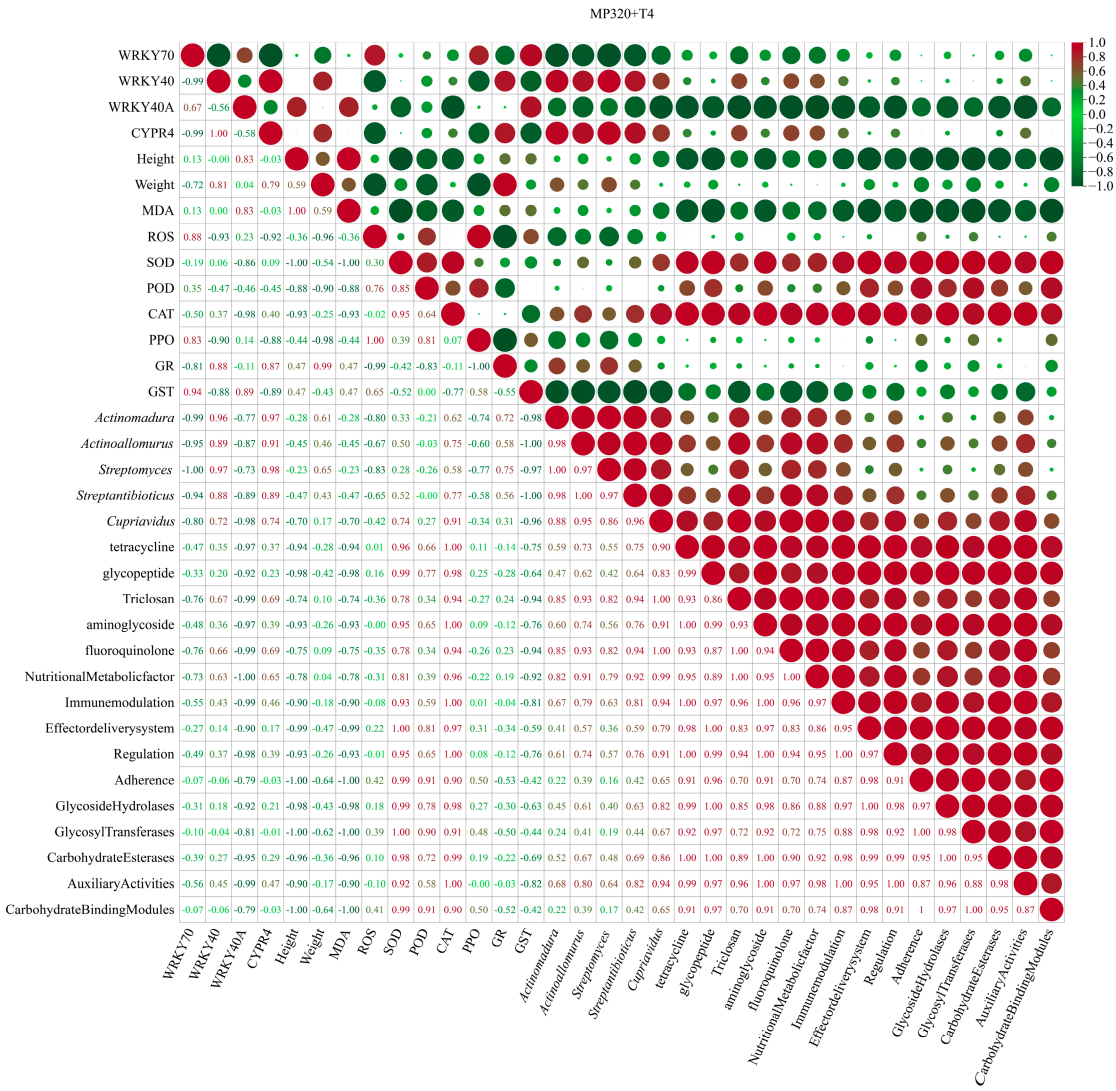

Supplementary Figure S7. Correlation analysis of *Nicotiana benthamiana* related indicators in the MP320+T4 group
